# Acetyl-11-keto-β-boswellic acid enhances the cisplatin sensitivity of non-small cell lung cancer cells through cell cycle arrest, apoptosis induction, and autophagy suppression via p21-dependent signaling pathway

**DOI:** 10.1007/s10565-020-09541-5

**Published:** 2020-06-20

**Authors:** Minghe Lv, Xibing Zhuang, Qi Zhang, Yunfeng Cheng, Duojiao Wu, Xiangdong Wang, Tiankui Qiao

**Affiliations:** Center for Tumor Diagnosis and Therapy, Jinshan Hospital, Fudan University, Jinshan District, Shanghai, 201508 China

**Keywords:** Acetyl-11-keto-β-boswellic acid, Cisplatin, Non-small cell lung cancer, Cell cycle, Apoptosis, Autophagy, p21

## Abstract

Cisplatin-based therapy is a widely used chemotherapeutic regimen for non-small cell lung cancer (NSCLC); however, drug resistance limits its efficacy. Acetyl-11-keto-β-boswellic acid (AKBA), a bioactive compound from frankincense, has been shown to exert anti-cancer effects. The aim of this study is to explore the potential of AKBA in combination with cisplatin as a new regimen for NSCLC. CCK8 assay and clone formation assay were used to determine the effects of AKBA in combination with cisplatin on cell viability of NSCLC cell lines. A three-dimensional spherification assay was used to simulate in vivo tumor formation. Flow cytometry was performed to examine cell cycle distribution and the percentages of apoptotic cells. The associated proteins and mRNA of cell cycle, apoptosis, and autophagy were measured by western blotting and real-time fluorescence quantitative PCR. Immunofluorescence assay was used to test apoptotic nuclei and autolysosome. Small interfering RNA experiments were used to silence the expression of p21. Combination treatment of AKBA and cisplatin inhibited cell viability, clone formation, and three-dimensional spherification, enhanced G_0_/G_1_ phase arrest, increased the percentages of apoptotic cells, and decreased the ratio of positive autolysosomes, compared with cisplatin alone. AKBA in combination with cisplatin suppressed the protein expressions of cyclin A2, cyclin E1, p-cdc2, CDK4, Bcl-xl, Atg5, and LC3A/B, and upregulated p27 and p21 mRNA levels in A549 cells. Downregulation of p21 decreased G_0_/G_1_ phase arrest and the percentages of apoptotic cells, and promoted autophagy in NSCLC A549 cells. Our study demonstrates that AKBA enhances the cisplatin sensitivity of NSCLC cells and that the mechanisms involve G_0_/G_1_ phase arrest, apoptosis induction, and autophagy suppression via targeting p21-dependent signaling pathway.

**Electronic supplementary material:**

The online version of this article (10.1007/s10565-020-09541-5) contains supplementary material, which is available to authorized users.

## Introduction

Lung cancer, the most common malignancies, is a leading cause of cancer-related death around the world (Bray et al. 2018). Non-small cell lung cancer (NSCLC) represents a most type of tumor, accounting for approximately 85% of new diagnosed lung cancer cases (Gridelli et al. 2015). The results of recent randomized trials with different cisplatin-based chemotherapy regimens have shown that platinum-based therapy is still the mainstay for treatment of NSCLC (Belani 2002). Cisplatin (CDDP) is a potent DNA-damaging and apoptosis induction chemotherapy agent (Matsumoto et al. 2016; Shamimi-Noori et al. 2008; Sun et al. 2018; Ulukaya et al. 2011). However, a high rate of relapse occurs following CDDP treatment despite the methods aiming to overcome resistance (Mitsudomi et al. 2010; Olaussen et al. 2006; Scagliotti et al. 2008), and chemotherapy resistance limited its curative effects in NSCLC patients. Therefore, it is of great significance to find new combined agents that increase the sensitivity of NSCLC cells to CDDP.

Natural plant ingredients have been used for clinical application in human culture. Many natural plant ingredients and their derivatives have been demonstrated be effective in treating tumors, such as camptothecin (Lazareva et al. 2018; Shamma and St 1974), paclitaxel (Meng et al. 2016; Rowinsky and Donehower 1995), and vinblastine (Silvestri 2013). Recent studies show that some of them have synergistic effects when combined with standard chemotherapeutic agents. For example, taxol, a unique tubulin active agent, was found to demonstrate a marked schedule-dependent synergistic interaction with CDDP in the killing of human ovarian carcinoma cells in vitro (Jekunen et al. 1994). Furthermore, nab-paclitaxel in combination with CDDP versus docetaxel plus CDDP as first-line therapy have been used to treat the advanced NSCLC patients (Chen et al. 2017). Compared with traditional chemotherapeutics, natural medicines are safer and cost less, making them ideal “chemosensitizers” for cancer management and treatment.

Acetyl-11-keto-β-boswellic acid (AKBA) is a pentacyclic triterpenes, which is the main component of boswellic acid from *Boswellia serrata*, extensively known as Indian olibanum that has been proved to reveal immense potential in combating cancer (Khan et al. 2016). Traditionally, boswellic acid has the effect of promoting blood circulation and removing wind, relieving muscle pain and swelling, widely used in the treatment of chronic inflammatory diseases (Ammon 2016), such as rheumatoid arthritis and osteoarthritis. As an anti-inflammatory agent, boswellic acid downregulates the TNF-α expression, suppresses the activity of active human recombinant GST-IKKα and His-IKKβ (Syrovets et al. 2005) and inhibits NF-ƙB and NF-ƙB-regulated gene expression (Takada et al. 2006). In the recent years, AKBA also has been found to exert anti-cancer effects in some tumors. For example, AKBA has been reported to inhibit prostate tumor growth by suppressing vascular endothelial growth factor receptor 2-mediated angiogenesis (X et al. 2009) and to suppress invasion of pancreatic cancer cells through the downregulation of CXCR4 chemokine receptor expression (Park et al. 2011). However, the effects of AKBA on NSCLC cells have rarely been reported, and whether or not AKBA can increase the chemo-sensitivity of CDDP in NSCLC and the underlying mechanisms is still unknown.

p21, a well-established cyclin-dependent kinase inhibitor (CKI), has been found to play an important role in regulating cell cycle progression (Harper et al. 1993), apoptosis (Eastham et al. 1995), and autophagy (Fujiwara et al. 2008). Recent studies indicate that targeting p21 regulators for therapy could be an effective way to prevent tumor growth and metastasis (Ji et al. 2017; Li et al. 2018a, b; Zhang et al. 2019). For example, exogenous expression of p21 (WAF1/CIP1) exerts cell growth inhibition and enhances the sensitivity of CDDP in hepatoma cells (Qin and Ng 2001). Therefore, targeting p21 pathway provides a promising therapeutic approach.

In this study, we aimed to evaluate the synergetic effects of AKBA in combination with CDDP on cell proliferation, cell cycle distribution, cell apoptosis, and cell autophagy in NSCLC cell lines. In addition, we attempted to explore the underlying mechanism that AKBA enhanced the sensitivity of CDDP in NSCLC cells.

## Materials and methods

### Reagents

Purified AKBA (> 98.0% pure) was purchased from Duma biotechnology company (Shanghai, China). It was dissolved in dimethyl sulfoxide (DMSO, Sigma, Louis, Missouri, USA) at 20 mg/ml and stored at − 20 °C until needed. The DMSO concentration of each treatment team was not more than 0.1%. Cisplatin (CDDP) was purchased from QILU Pharmaceutical (Shandong, China), and was dissolved in phosphate buffer solution (PBS, Keygen Biotech, Nanjing, China) at 0.5 mg/ml. CDDP was also stored at −20 °C until needed.

### Cell lines and cell culture

The human NSCLC cell line A549 was obtained from the Cell Bank of the China Science Academy (Shanghai, China). The normal human lung epithelium cell line BEAS-2B and the human NSCLC cell lines H460 and H1299 were purchased from Cell Research (Shanghai, China). A549, H460, and H1299 were maintained in RPMI-1640 medium (Sigma, Louis, Missouri, USA) contained 10% Fetal bovine serum (Biological Industries, Israel). All cells were cultured at 37 °C under 5% CO_2_. BEAS-2B was cultured with complete medium for bronchial epithelial cells (Cell Research, Shanghai, China).

### Three-dimensional spherification assay

The human NSCLC cell line A549 cells (5 × 10^3^ cells) were seeded into 96-well plates that were coated with 1% agar gel (Sigma-Aldrich; Merck KGaA) which prevents cell attachment and results in the cells’ suspension aggregating into cell spheroids. Then, the growth characteristics of spheroids were observed and recorded by an inverted microscope every day (IX73; Olympus Corporation, Tokyo, Japan). After a month of cultivation, cells of each group were calculated by hemocytometer.

### Cell viability assay

Cells were seeded into 96-well plates at a density of 5000 cells per well and incubated for 24 h. Then, cells were treated with AKBA (10 μg/ml) or CDDP (0, 1, 2, 4, and 8 μg/ml) or AKBA (10 μg/ml) plus CDDP (2 μg/ml) for 24 h,48 h, and 72 h. Cell viability was determined by widely accepted assay, Cell Counting Kit-8 Assay Kit (Do Jindo Laboratories, Kumamoto, Japan). The experiments were conducted according to the manufacturer’s protocol and were performed in triplicate.

### Colony formation assay

A549 and H1299 cells were seeded into 6-well plates at 2 × 10^5^ cells per well. Then, cells were treated with AKBA (10 μg/ml), CDDP (2 μg/ml), or AKBA (10 μg/ml) plus CDDP (2 μg/ml) for 48 h. Then, cells will be digested with trypsin (Keygen Biotech, Nanjing, China), and 500 cells of each group were reseeded to 6-well plates and continually cultured for 8–10 days. Finally, the colonies were stained with crystal violet staining solution (Sigma, Louis, Missouri, USA).

### Cell cycle analysis

To determine the cell cycle distribution, about 2 × 10^5^ cells were collected in flow tube after treatment and fixed in 70% ethyl alcohol at − 20 °C overnight. Then, the cells were washed with PBS three times and incubated with 0.5 ml PI/RNase Staining Buffer (BD Biosciences, Franklin, NJ, USA) for 15 min. The fractions of the cells in G0/G1, S, and G2/M phase were analyzed by flow cytometry (Beckman Coulter or BD Biosciences, USA).

### Apoptosis analysis

Cells were treated with AKBA (10 μg/ml), CDDP (2 μg/ml), or AKBA (10 μg/ml) plus CDDP (2 μg/ml) for 48 h. Apoptotic cells were determined by Annexin V-FITC Apoptosis Detection Kit (Do Jindo Laboratories, Kumamoto, Japan), according to the manufacturer’s protocol. Cell apoptosis was analyzed by flow cytometry (Beckman Coulter or BD Biosciences, USA).

### Western blot analysis

After treatment, the liquid supernatant was taken out and the cells were washed thrice in the surface of 6-well plates. Then, cell lysates were prepared and the concentration of protein was quantified by using BCA protein assay kit (ThermoFisher, USA). Equal amount of proteins was subjected to 10% or 12% SDS-polyacrylamide gel electrophoresis and transferred to PVDF membranes (Millipore, Bedford, MA, USA). Membranes were blocked and probed with specific antibodies (dilution ratio: 1:3000, anti-cyclin A2, anti-cyclin E1, anti-cyclin D2, anti-p-cdc2, anti-CDK2, anti-CDK4, anti-Bax, anti-Bcl-xl, anti-p62, anti-Atg-5, anti-Beclin-1, anti-LC3A/B, and anti-β-actin, Cell Signaling Technology, Danvers, MA, USA) followed by exposure to a horseradish peroxidase–conjugated goat anti-mouse or goat anti-rabbit antibody and secondary antibodies (dilution ratio: 1:5000, Cell Signaling Technology, Danvers, MA, USA). The immunocomplexes were visualized using a horseradish peroxidase-conjugated antibody, followed by a chemiluminescence reagent (Millipore, Bedford, MA, USA) and detected on photographic film.

### Immunofluorescence

After treatment, nuclear staining of cells was performed with 2 μg/ml 4′,6-diamidino-2-phenylindole (DAPI, Keygen Biotech, Nanjing, China) staining solution at 37 °C for 10 min. DAPI can attach to the minor groove of double-stranded DNA, forming a stable compound with fluorescence enhancement. To detect the formation of autolysosome, the cells were seeded into 24-well plates at 2 × 10^4^ per well, and treated with AKBA for 48 h. Then, cells were washed with RPMI-1640 medium (Sigma, Louis, Missouri, USA) and treated with DALGreen Working Solution (Do Jindo Laboratories, Kumamoto, Japan) for 30 min. After the treatment with DALGreen Working Solution, cells were washed with RPMI-1640 medium again and cultured with autophagy inducer rapamycin (Ji Kai Gene Technology Company, Shanghai, China) for 24 h. The cells were observed under a fluorescent microscope (IX73; Olympus Corporation). Cells with more than three DALGreen-positive foci were considered to be positive autophagy cells.

### Reverse transcription and polymerase chain reaction

RNA isolation was performed using the RNA Purification Kit (Yi Shan Biotechnology Company, Shanghai, China). cDNA was prepared by using the 5 × Reverse Transcription Master Mix (Takara, Osaka, Japan) and was performed according to the manufacturer’s protocol. Primers used in these experiments were as follows: β-actin, forward 5′-CATTGCCGACAGGATGCAG-3′, reverse 5′-CTCGTCATACTCCTGCTTGCTG-3′; P21, forward 5′-CATGTGGACCTGTCACTGTCTTGTA-3′, reverse 5′-GAAGATCAGCCGGCGTTG-3′; P27, forward 5′-CAATGCCGGTTCTGTGGAG-3′, reverse 5′-TCCATTCCATGAAGTCAGCGATA-3′. After reverse transcription, the cDNA product was amplified by PCR according to the manufacturer’s protocol (Takara, Osaka, Japan) and gene expression was quantified according to the 2^−ΔCt^ method.

### Small interfering RNA transfection

Lipofectamine 2000 reagents (Invitrogen, Carlsbad, CA, USA) were used for siRNA transfection according to the manufacturer’s instructions. P21 siRNA and NC siRNA were purchased from Gemma Pharma (Shanghai, China) and the specific siRNA sequences were as following:

P21-homo-376, sense (5′–3′): GAUGGAACUUCGACUUUGUTT, antisense (5′–3′): ACAAAGUCGAAGUUCCAUCTT; P21-homo-887, sense (5′–3′): CCUCUGGCAUUAGAAUUAUTT, antisense (5′–3′): AUAAUUCUAAUGCCAGAGGTT; P21-homo-1120, sense (5′–3′): CAGGCGGUUAUGAAAUUCATT, antisense (5′–3′): UGAAUUUCAUAACCGCCUGTT; NC, sense (5′–3′): UUCUCCGAACGUGUCACGUTT; antisense (5′–3′): ACGUGACACGUUCGGAGAATT; The effect of gene silencing on protein level was measured by western blot at 48 h after transfection.

### Statistical analysis

The statistical data were analyzed by GraphPad Prism 7 (GraphPad Software, San Diego, CA, USA) and Microsoft Office Excel 2017 (Microsoft Corporation, Redmond, WA, USA). Student’s *t* test was used to determine differences between the two compared groups. Statistical significance (*P* < 0.05) was determined by one-way analysis of variance (ANOVA) and Tukey’s test among 3 or more groups. All statistical results are expressed as the mean ± SD. All the experiments were performed at least three times.

## Results

### AKBA enhanced the inhibition effects of CDDP on cell viability in human NSCLC cell lines, but weakened the suppression effects of CDDP on cell viability in BEAS-2B cells

**Fig. 1:**
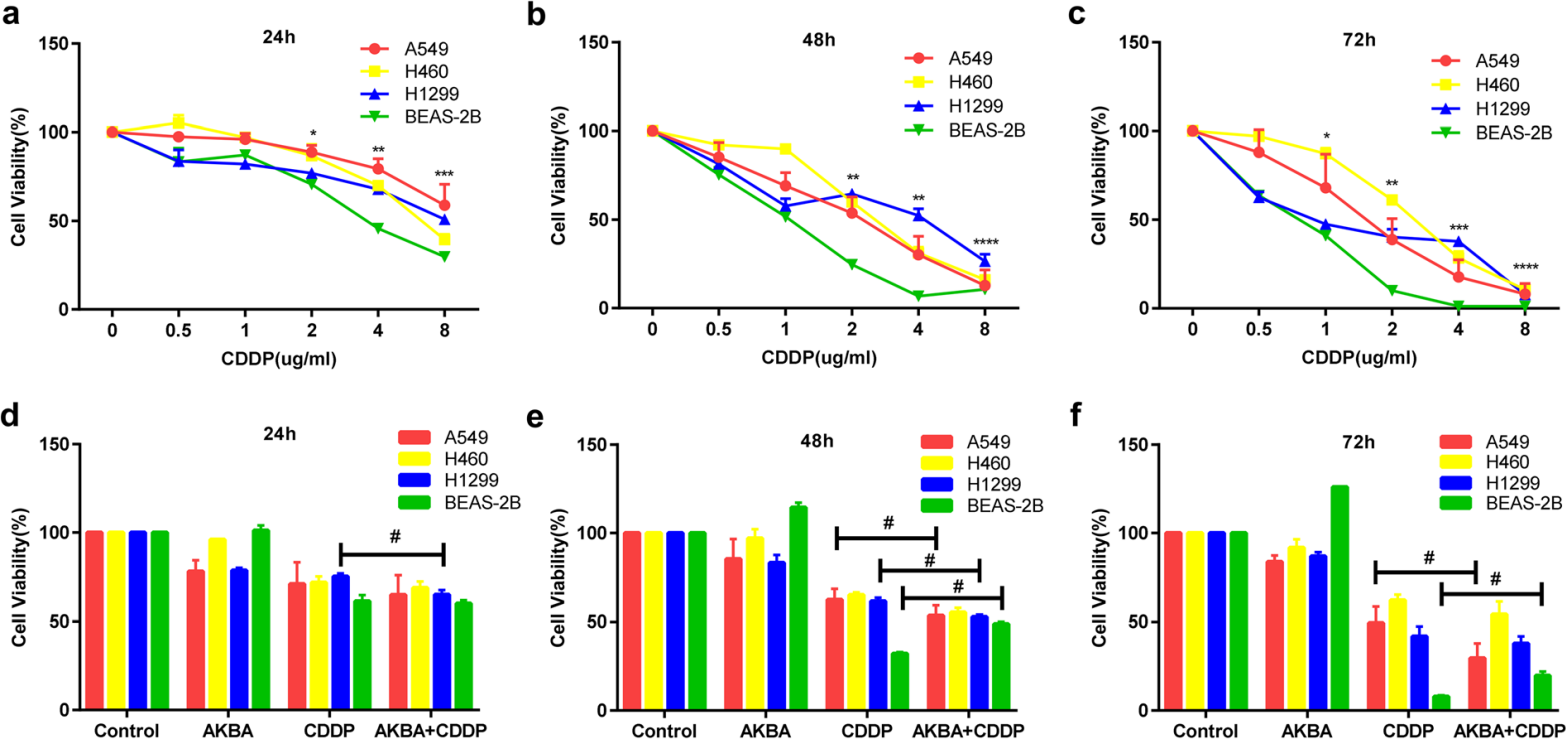
The effects of CDDP in combination with AKBA on cell viability of A549, H460, H1299, and BEAS-2B. **a**, **b**, **c** CCK8 assay showed that CDDP reduced the cell viability of A549, H460, H1299, and BEAS-2B at 24 h, 48 h, and 72 h. **d**, **e**, **f** A549, H460, H1299, and BEAS-2B were treated with AKBA (10 μg/ml), CDDP (2 μg/ml), or AKBA (10 μg/ml) + CDDP (2 μg/ml) for 24 h, 48 h, and 72 h, and the cell viability was determined by CCK8 assay. Data were represented as the mean ± SD of 3 independent experiments, *P < 0.05, **P < 0.01, ***P < 0.001, ****P < 0.0001, vs. the CDDP-untreated control group (0 μg/ml). ^#^*P* < 0.05, between CDDP group and the combination group

CDDP reduced the cell viability of human NSCLC cell lines (A549, H460, and H1299) and BEAS-2B in time- and dose-dependent manner **(**Fig. 1a–c**)**. Fifty percent inhibitory concentration (IC_50_) values for CDDP on A549 cells at 24 h, 48 h, and 72 h were 10.65 μg/ml, 2.05 μg/ml, and 1.57 μg/ml. IC_50_ values for CDDP on H460 cells at 24 h, 48 h, and 72 h were 6.3 μg/ml, 2.661 μg/ml, and 2.544 μg/ml. IC_50_ values for CDDP on H1299 cells at 24 h, 48 h, and 72 h were 10.24 μg/ml, 3.07 μg/ml, and 1.042 μg/ml. IC_50_ values for CDDP on BEAS-2B at 24 h, 48 h, and 72 h were 3.808 μg/ml, 1.019 μg/ml, and 0.7296 μg/ml. According to IC_50_ values of AKBA in the previous study (Lv et al. 2020) and CDDP on cell viability, as well as the effects of AKBA at 5 μg/ml, 10 μg/ml, and 15 μg/ml in combination with CDDP on human NSCLC cell lines and BEAS-2B in this study (Fig. S2a, b, c), we choose appropriate combined concentration (AKBA 10 μg/ml, CDDP 2 μg/ml) to treat cell lines. After 24 h of treatment, we found that AKBA enhanced significantly the repressive effect of CDDP in H1299, but not in A549, H460, and BEAS-2B (Fig. 1d). In A549 and H1299, CDDP in combination with AKBA reduced markedly the cell viability at 48 h, compared with CDDP alone. However, in BEAS-2B, the combination of CDDP with AKBA increased obviously the cell viability at 48 h, compared with CDDP alone (Fig. 1e). As shown in Fig. 1f, AKBA enhanced the cell viability suppression effects of CDDP in A549 cells at 72 h, but not in H460 and H1299 cells. By contrast, AKBA weakened the cell viability inhibition effects of CDDP in BEAS-2B at 72 h, which suggested that AKBA could enhance the inhibitory effects of CDDP on NSCLC cell lines and protect normal bronchopulmonary epithelial cells (BEAS-2B) treated with CDDP.

### AKBA strengthened the suppression effects of CDDP on clone formation in A549 and H1299 cells

**Fig. 2:**
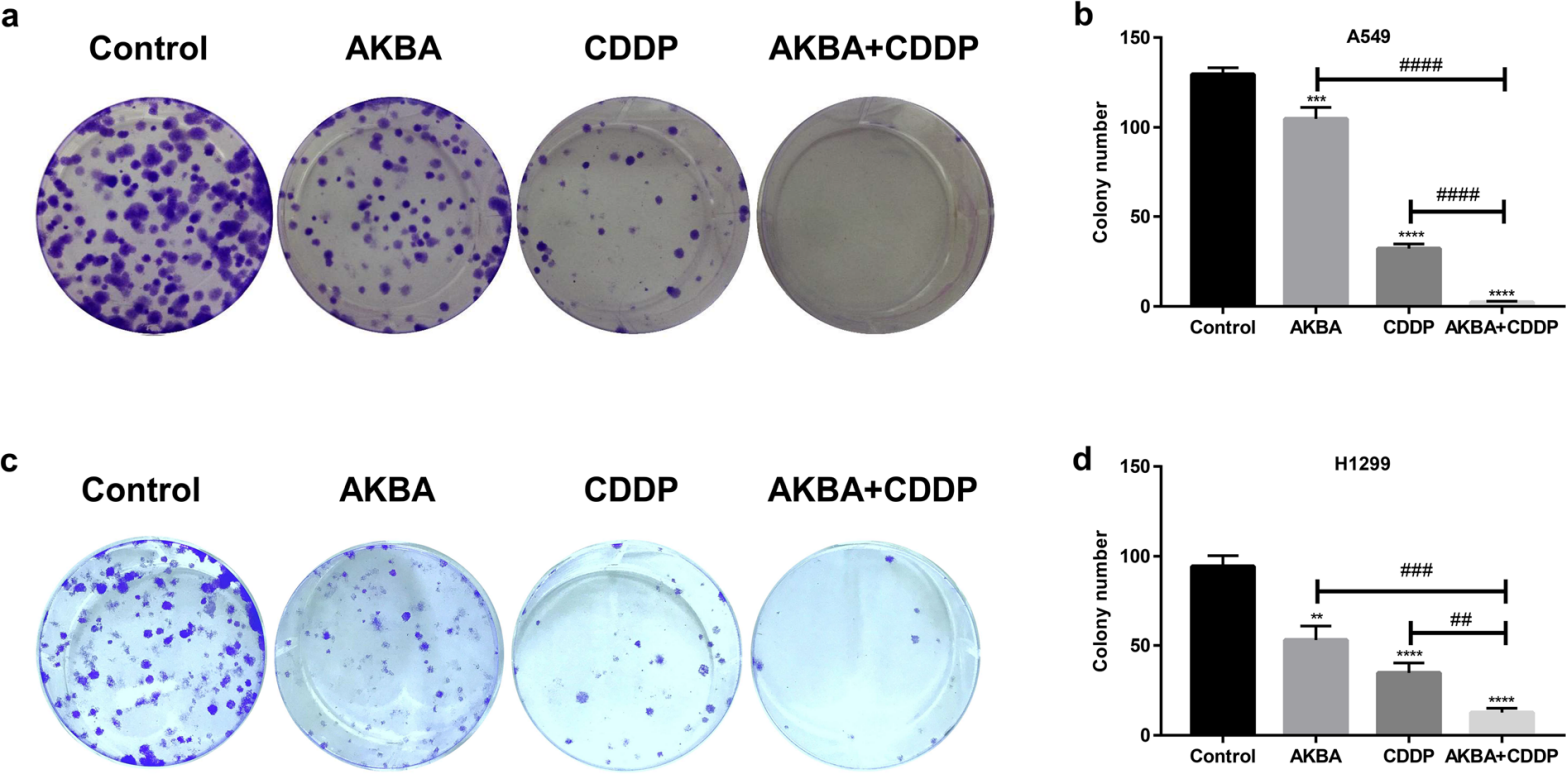
The effects of CDDP in combination with AKBA (CDDP 2 μg/ml, AKBA 10 μg/ml) on clone formation of A549 and H1299 cells. **a** The combination of CDDP with AKBA enhanced the suppression effects of clone formation in A549 cells. **b** Histogram showing the clone number in A549 cells and relative statistical analysis. **c** The combination of CDDP with AKBA enhanced the suppression effects of clone formation in H1299 cells. **d** Histogram showing the clone number in H1299 cells and relative statistical analysis. Data were represented as the mean ± SD of 3 independent experiments, **P < 0.01, ***P < 0.001, ****P < 0.0001, vs. control group. ^##^*P* < 0.01, ^###^*P* < 0.001, ^####^*P* < 0.0001

As shown in Fig. 2a, the proliferation ability of A549 cells was determined by clone formation assay. We found that AKBA in combination with CDDP reduced significantly the clone number of A549 cells, compared with AKBA or CDDP alone (Fig. 2b). In addition, after cotreatment with AKBA and CDDP in H1299 cells, we found that the clone number were reduced obviously, compared with treatment of AKBA or CDDP alone (Fig. 2c, d). Therefore, this part of data indicated that AKBA enhanced the sensitivity of CDDP to NSCLC cells by suppressing cell proliferation. Moreover, we also detected the colony formation of BEAS-2B after cotreatment of AKBA and CDDP, finding that AKBA increased the colony number of BEAS-2B compared to control group (Fig. S3a, b).

### Effects of cotreatment with AKBA and CDDP on a three-dimensional spherification and cell morphology

**Fig. 3:**
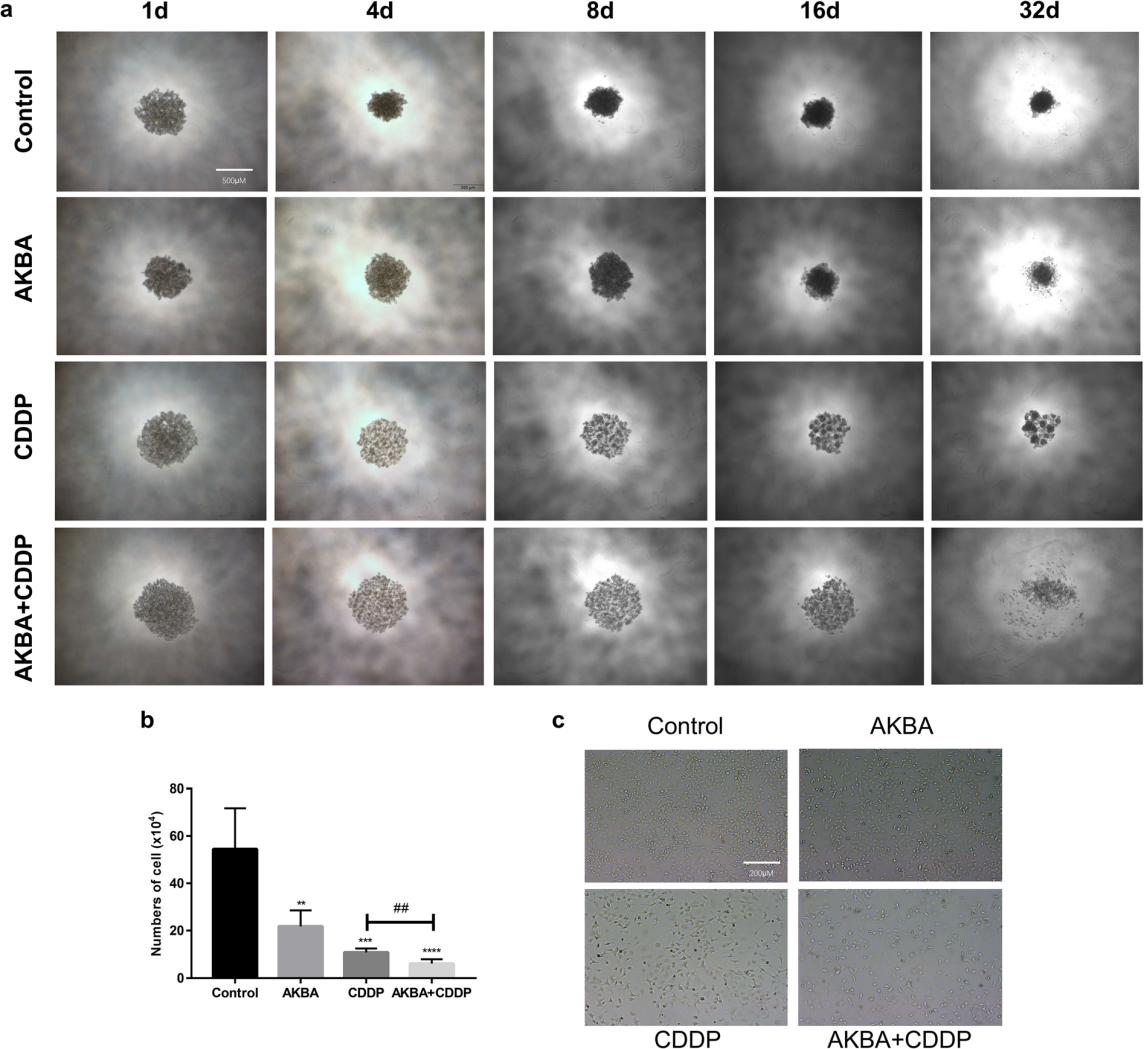
Effects of AKBA in combination with CDDP (AKBA 10 μg/ml, CDDP 2 μg/ml) on a three-dimensional spherification and cell morphology. **a** Representative graph of a three-dimensional spherification assay after cotreatment with AKBA and CDDP in A549 cells. Scale bar = 500 μM. **b** Histogram showing the cell numbers of A549 of cotreatment with AKBA and CDDP on the 32nd day and relative statistical analysis. **c** Morphology change of A549 after cotreatment with AKBA and CDDP. Scale bar = 200 μM. Data were represented as the mean ± SD of 3 independent experiments, **P < 0.01, ***P < 0.001, ****P < 0.0001, vs. control group. ^##^*P* < 0.01

A549 cells (5 × 10^3^) were cultured in agar gel-coated 96-well plates and treated with AKBA, CDDP alone, or in combination. The growth characteristics of A549 were recorded by using an inverted microscope every 2 days. As shown in Fig. 3a, we found that the density of three-dimensional spheres formed by A549 cells in the control group were increased over time; however, the sphere density of AKBA, CDDP, or AKBA plus CDDP groups were markedly lower than control group at the same time. In particular, we found that the sphere structure of AKBA plus CDDP group was significantly destructed compared with CDDP alone group after 32 days, which suggested that AKBA in combination CDDP could exert more suppression effects on cell proliferation. Moreover, statistical analysis of cell count showed that AKBA combined with CDDP was more effective in inhibiting pellet formation in A549 cells on the 32nd day (Fig. 3b). In this study, we also found that treatment of AKBA, CDDP alone, or in combination all could alter the morphology of A549, and also reduced the cell number compared with control group (Fig. 3c). Therefore, these evidences demonstrated that AKBA could enhance the sensitivity of CDDP on cell proliferation.

### AKBA enhanced the sensitivity of CDDP to A549 via arresting cell cycle at G_0_/G_1_ phase

**Fig. 4:**
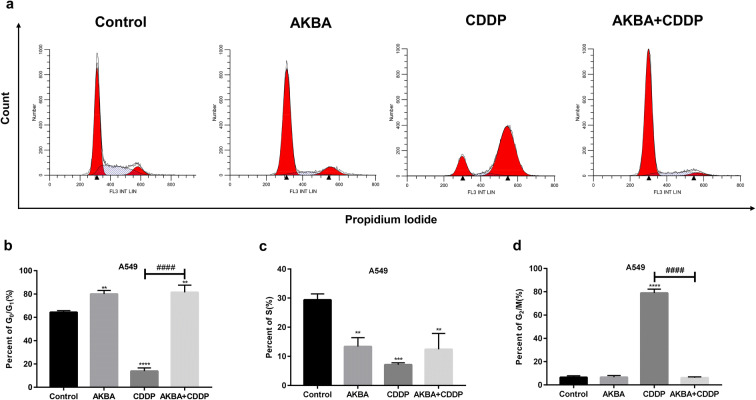
AKBA enhanced the sensitivity of A549 cells to CDDP via arresting cell cycle at G_0_/G_1_ phase. **a** A549 cells were treated with AKBA (10 μg/ml), CDDP (2 μg/ml), or AKBA (10 μg/ml) + CDDP (2 μg/ml) for 48 h, then the cell cycle distribution was measured by flow cytometry. **b** Histogram showing the cell cycle of A549 cells in G_0_/G_1_ phase and relative statistical analysis. **c** Histogram showing the cell cycle of A549 cells in S phase in each group and relative statistical analysis. **d** Histogram showing the cell cycle of A549 cells in G_2_/M phase and relative statistical analysis. Data were represented as the mean ± SD of 3 independent experiments, **P < 0.01, ***P < 0.001, ****P < 0.0001, vs. control group. ^####^*P* < 0.0001

After the cotreatment with AKBA and CDDP for 48 h, the cell cycle distribution was measured by flow cytometry. As shown in Fig. 4a, AKBA arrested the cell cycle at G_0_/G_1_ phase in A549, but CDDP increased distinctly the percentages of G_2_/M phase. Interestingly, after cotreatment of AKBA with CDDP to A549, the percentages of G_0_/G_1_ phase were increased and the frequencies of G_2_/M phase were reduced, compared with CDDP alone. In this study, we found that AKBA enhanced the sensitivity of CDDP to A549 via increasing clearly the percentages of G_0_/G_1_ phase (Fig. 4b–d).

### Effects of combination treatment of AKBA and CDDP on cell cycle regulators in A549 cells

**Fig. 5:**
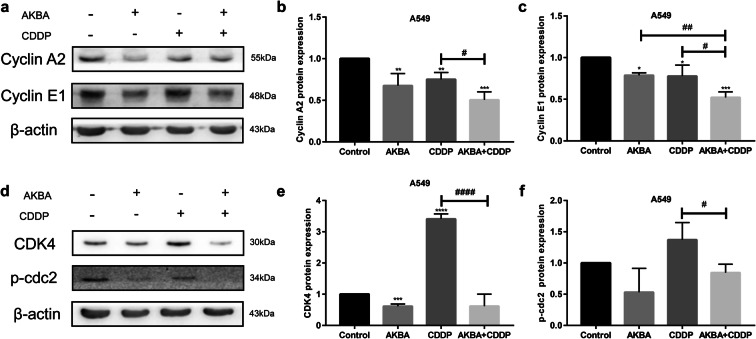
Effects of cotreatment with AKBA and CDDP (AKBA 10 μg/ml, CDDP 2 μg/ml) on cell cycle regulators. **a** The expressions of cyclin A2 and cyclin E1 proteins were determined by western blotting assay in the four groups. **b** Histogram showing the level of cyclin A2 protein expression and relative statistical analysis. **c** Histogram showing the level of cyclin E1 protein expression and relative statistical analysis. **d** Representative images of CDK4 and p-cdc2 proteins, using β-actin as internal control. **e** Histogram showing the level of CDK4 protein expression and relative statistical analysis. **f** Histogram showing the level of p-cdc2 protein expression and relative statistical analysis. Data were represented as the mean ± SD of 3 independent experiments, **P < 0.01, ***P < 0.001, ****P < 0.0001, vs. control group. ^#^*P* < 0.05, ^##^*P* < 0.01, ^####^*P* < 0.0001

The protein expressions of cyclin A2 and cyclin E1 were measured by western blotting, using β-actin as internal reference (Fig. 5a). In this study, we found that cyclin A2 and cyclin E1 protein expression levels were downregulated by AKBA or CDDP alone, and they were further decreased after the treatment of CDDP combined with AKBA (Fig. 5b, c). Furthermore, we examined the expression of CDK4 and p-cdc2 proteins by using western blotting assay (Fig. 5d). As shown in Fig. 5e, the expression of CDK4 protein was decreased by treatment of AKBA but was increased by treatment of CDDP in A549; however, it was decreased by synergetic treatment of AKBA and CDDP, compared with CDDP alone. We also found that AKBA in combination with CDDP amplified the inhibition effects of p-cdc2 protein expression compared with CDDP alone (Fig. 5f). Additionally, the expressions of cyclin A2 and p-cdc2 proteins were decreased by treatment of CDDP but were elevated by treatment of AKBA in combination with CDDP (Fig. S4a, b, c).

### AKBA in combination with CDDP increased the mRNA and protein expression levels of p27 and p21 in A549 cells

**Fig. 6:**
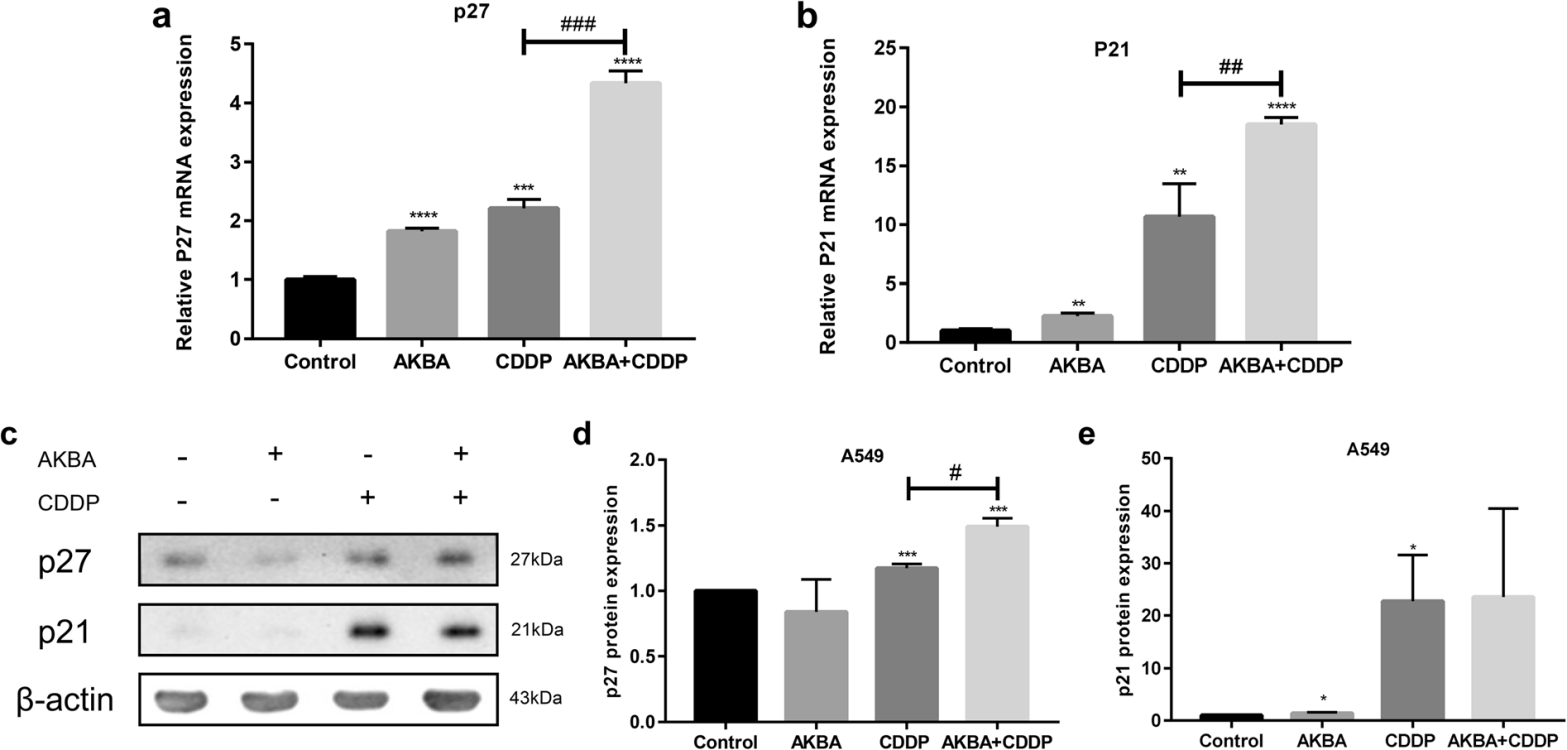
Effects of cotreatment with AKBA and CDDP (AKBA 10 μg/ml, CDDP 2 μg/ml) on the mRNA and protein expression levels of p27 and p21. **a** The mRNA expression level of p27 was measured by PCR in each group. **b** The mRNA expression level of p21 was measured by PCR in each group. **c** The protein expression levels of p27 and p21 were measured by western blotting assay in each group. **d, e** Histogram showing the levels of p27 and p21 protein expression and relative statistical analysis. Data were represented as the mean ± SD of 3 independent experiments, *P < 0.05, **P < 0.01, *** *P* < 0.001, ****P < 0.0001, vs. control group. ^#^*P* < 0.05, ^##^*P* < 0.01, ^###^*P* < 0.001

Cyclin-dependent kinase inhibitors (CKI), as the upstream regulators of cell cycle proteins, play a vital role in regulating negatively cell cycle. In this study, we employed real-time fluorescent quantitative PCR to examine the mRNA expression levels of p27 and p21, finding that combination treatment of AKBA and CDDP upregulated visibly the mRNA expression of p27 and p21 compared with CDDP alone in A549 cells (Fig. 6a, b). To further investigate the function effects of p21 and p27 after cotreatment with AKBA and CDDP, we used western blotting assay to examine the protein expression of p27 and p21, finding that AKBA in combination with CDDP strengthened the expression of p27 protein, but rarely increased the expression of p21 protein, compared with CDDP alone (Fig. 6c–e). This part of data possibly suggested that AKBA enhanced the sensitivity of CDDP on cell cycle via upregulating the gene expressions of p27 and p21, then suppressing the cyclical proteins expression. In addition, we also detected the expression levels of p27 and p21 proteins in BEAS-2B cells, finding that the expressions of p27 and p21 were decreased by treatment of CDDP but were elevated by treatment of AKBA in combination with CDDP (Fig. S4d, e, f).

### AKBA enhanced the sensitivity of CDDP to A549 via increasing apoptosis

**Fig. 7:**
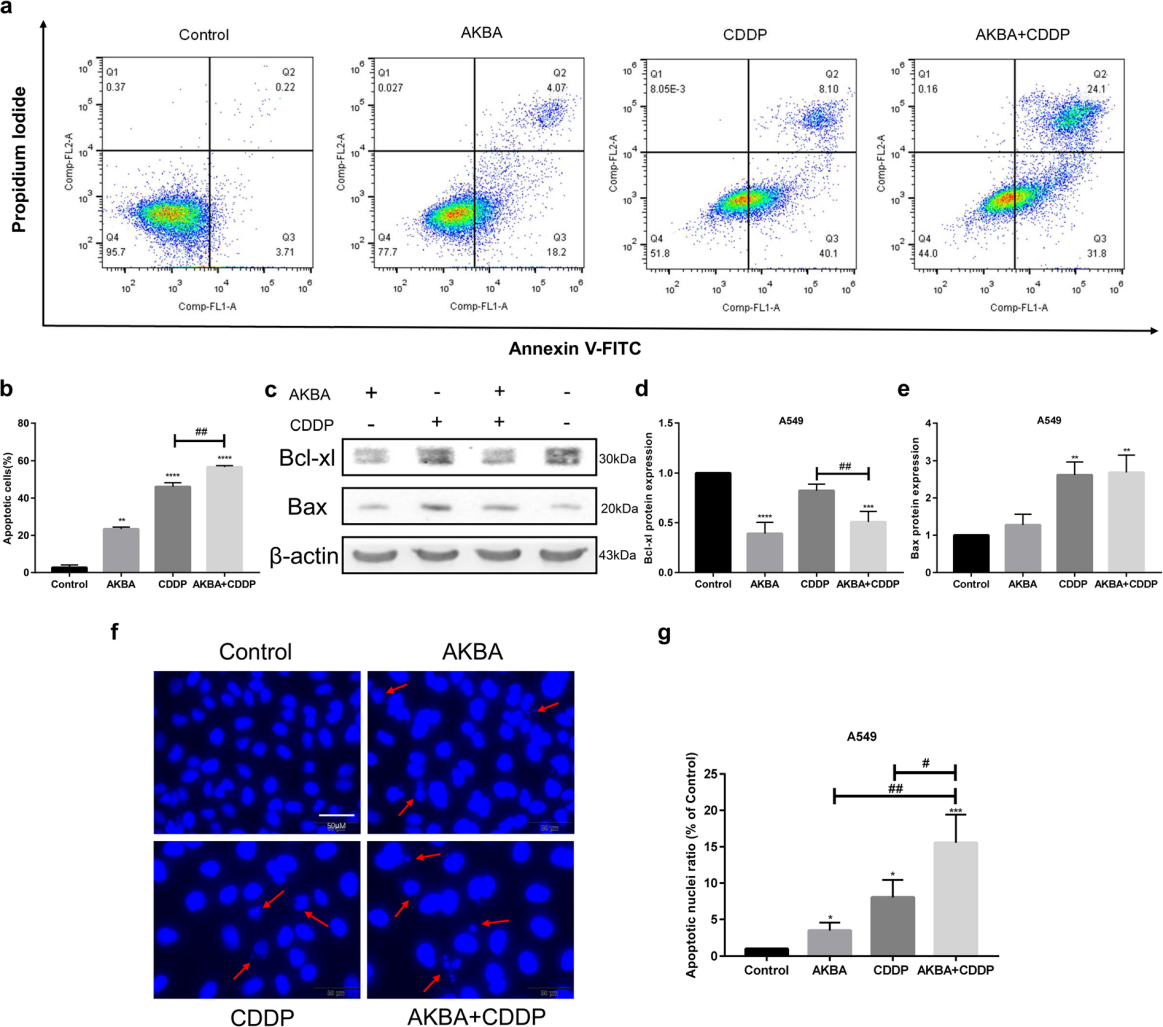
AKBA strengthened the sensitivity of CDDP (AKBA 10 μg/ml, CDDP 2 μg/ml) through inducing apoptosis in A549 cells. **a** Flow cytometry showed that AKBA in combination with CDDP enhanced the apoptotic cell percentages in A549 cells. **b** Histogram showing the apoptotic cell percentages in each group. **c** Representative graphs of Bcl-xl and Bax protein expressions after cotreatment with AKBA and CDDP in A549 cells. **d**, **e** Histogram showing the expressions of Bcl-xl and Bax proteins. **f**, **g** Morphological alterations of the apoptotic nuclei of A549 in each group and relative statistical analysis. Scale bar = 50 μM. Data were represented as the mean ± SD of 3 independent experiments, *P < 0.05, **P < 0.01, ***P < 0.001, ****P < 0.0001, vs. control group. ^#^*P* < 0.05, ^##^*P* < 0.01

In this study, we used flow cytometry to measure the percentages of apoptotic cells in A549 cells after the treatment of AKBA, CDDP alone, or AKBA plus CDDP (Fig. 7a). As shown in Fig. 7b, the proportions of apoptotic cells were increased by AKBA and CDDP, and they were further elevated by synergetic therapy of AKBA and CDDP. In addition, to further explore the cotreatment effects of AKBA and CDDP on apoptosis, the apoptosis-related proteins were determined by western blotting assay in A549 cells (Fig. 7c). Bcl-xl, as an anti-apoptotic protein, was decreased after cotreatment of AKBA and CDDP, compared with CDDP alone, which suggested that AKBA could enhanced the effects of CDDP on apoptosis in A549. We also found that compared with control group, the expression of pro-apoptotic protein Bax was increased after treatment of AKBA, CDDP alone, or AKBA in combination with CDDP in A549 cells **(**Fig. 7d, e**)**. To further explore the effects of combination of AKBA and CDDP on apoptosis, we employed DAPI staining assay to examine the morphological alterations of the apoptotic nuclei (the red arrow pointed to a nucleus that had either shrunk or broken), finding that compared with AKBA or CDDP alone, AKBA in combination with CDDP could significantly cause the contraction and rupture of nucleus and increased the ratio of apoptotic nuclei in A549 cells (Fig. 7f, g).

### AKBA enhanced the sensitivity of CDDP to A549 via inhibiting autophagy

**Fig. 8:**
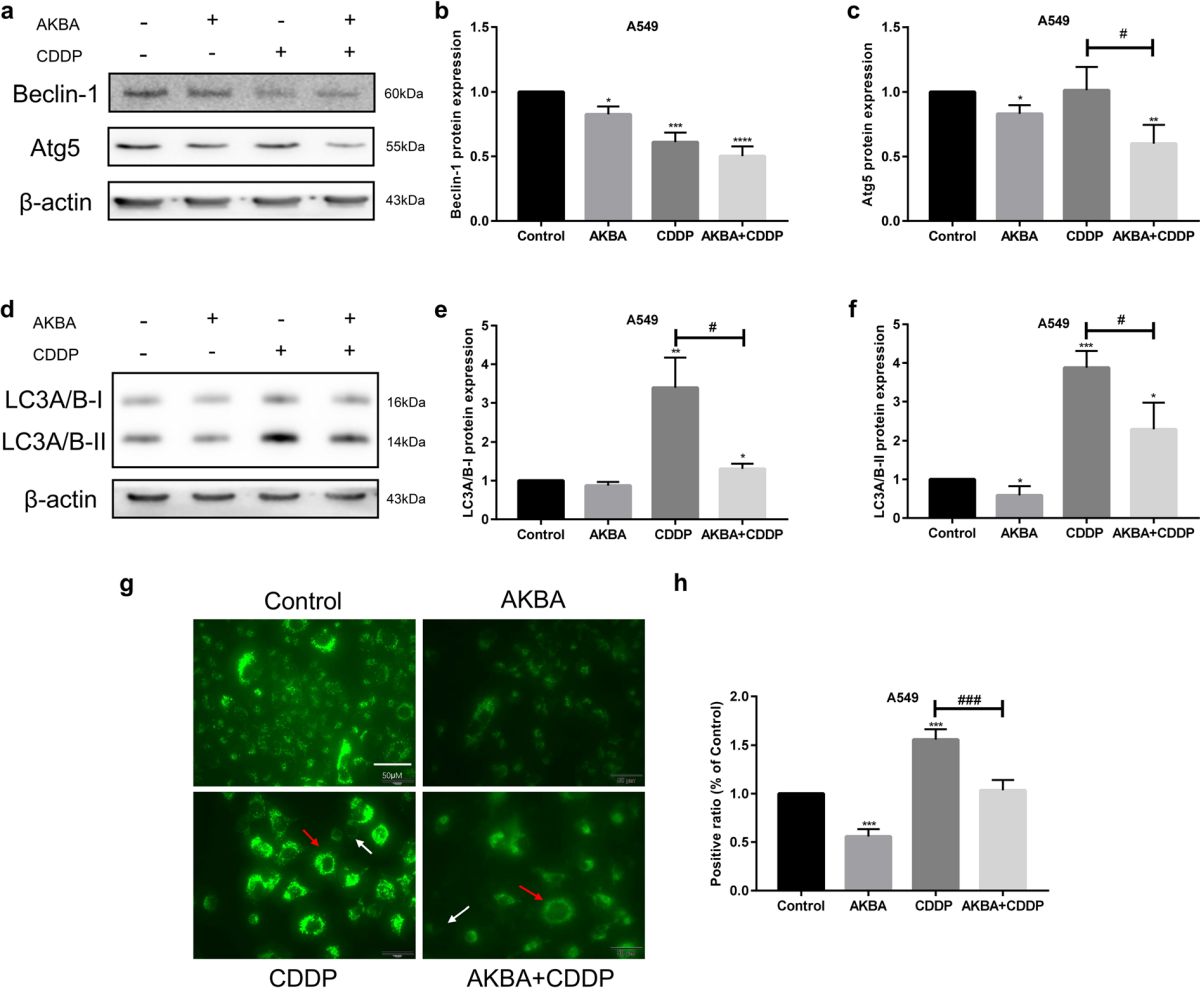
AKBA enhanced the sensitivity of CDDP (AKBA 10 μg/ml, CDDP 2 μg/ml) by suppressing autophagy. **a** The protein expression levels of Beclin-1 and Atg5 were measured by western blotting assay in each group. **b**, **c** Histogram showing the level of Beclin-1 and Atg5 proteins and relative statistical analysis. **d** Representative images of LC3A/B-I and LC3A/B-II protein expressions. **e**, **f** Histogram showing the level of LC3A/B-I and LC3A/B-II proteins and relative statistical analysis. **g** The formation of autolysosome was measured by immunofluorescence in A549 cells. White arrow indicates negative autolysosome cell and the red indicates positive autolysosome cell. **h** Quantification of the positive phagosomes in A549. Scale bar = 50 μM. Data were represented as the mean ± SD of 3 independent experiments, *P < 0.05, **P < 0.01, ***P < 0.001, vs. control group. ^#^*P* < 0.05, ^###^*P* < 0.001

To detect the effects of AKBA and CDDP on autophagy, the expression of Beclin-1 and Atg5 proteins was determined by western blotting assay in A549, using β-actin as internal reference (Fig. 8a). Statistical analysis of Beclin-1 expression level showed that AKBA in combination with CDDP decreased the protein expression of Beclin-1 in A549 cells (Fig. 8b). We found that the expression of Atg5 was decreased by treatment of AKBA, but was increased after treated with CDDP, and it was reduced when combined CDDP with AKBA in A549 cells (Fig. 8c). LC3A/B, a protein marker of autophagy, was detected by western blotting in A549, using β-actin as internal reference (Fig. 8d). After the treatment of AKBA, the expressions of LC3A/B-I and LC3A/B-II proteins were decreased, but they were increased by treatment of CDDP. However, AKBA in combination with CDDP reduced significantly the expression levels of LC3A/B-I and LC3A/B-II proteins in A549 cells, compared with CDDP alone (Fig. 8e, f). In addition, we used immunofluorescence technique to observe the formation of autolysosome under an inverted microscope, showing that AKBA inhibited the formation of positive autolysosome and reduced the fluorescence intensity compared with control group, CDDP induced autophagy because it increased the ratio of positive autolysosome and made fluorescence intense, and the combination treatment of two drugs made fluorescence dim and decreased the formation of positive autolysosome compared with CDDP alone **(**Fig. 8g, h).

### AKBA enhanced the sensitivity of CDDP through G_0_/G_1_ phase arrest via p21-dependent signaling pathway in A549 cells

**Fig. 9:**
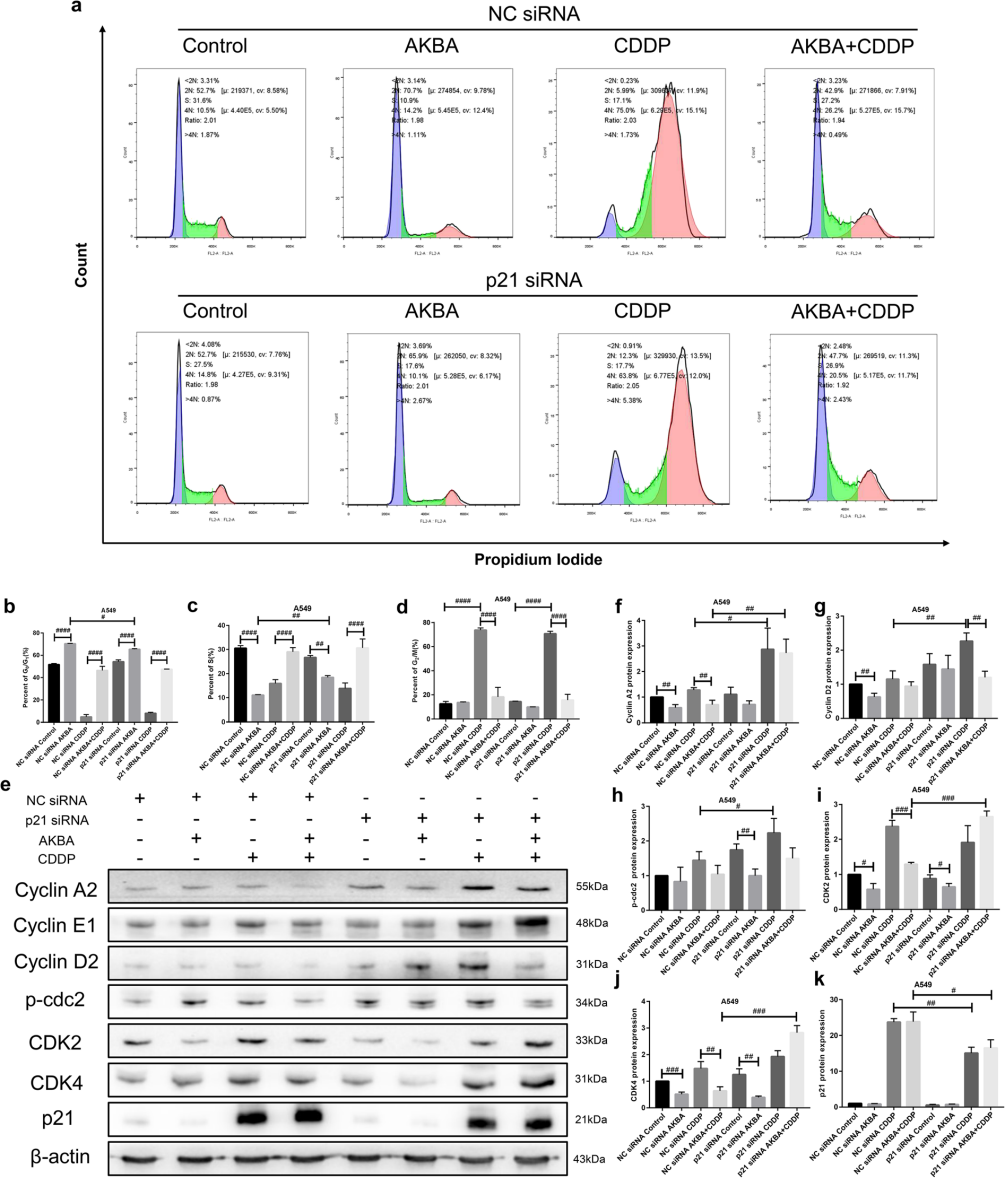
AKBA enhanced the sensitivity of CDDP (AKBA 10 μg/ml, CDDP 2 μg/ml) through G_0_/G_1_ phase arrest via p21-dependent signaling pathway in A549 cells. **a** Flow cytometry showed the cell cycle distribution of cotreatment with AKBA and CDDP in NC siRNA and p21 siRNA groups. **b**–**d** Histogram showing the of percentages of G_0_/G_1_, S, and G_2_/M phase in A549 cells treated with NC siRNA or p21 siRNA and relative statistical analysis. **e** Representative graphs of western blotting of AKBA in combination with CDDP after transfection of NC siRNA or p21 siRNA in A549 cells. **f**–**k** Histogram showing the level of cyclin A2, cyclin D2, p-cdc2, CDK2, CDK4, and p21 proteins and relative statistical analysis. Data were represented as the mean ± SD of 3 independent experiments. ^#^*P* < 0.05, ^##^*P* < 0.01, ^###^*P* < 0.001, ^####^*P* < 0.0001

To further explore the anti-tumor mechanism of cotreatment with AKBA and CDDP, we transfected three different p21 small interfering RNAs (siRNA) into A549 cells and selected the p21 siRNA with the best knockdown effect for subsequent experiments (Fig. S1). In this part, to detect the function effects of p21 on cell cycle in A549 cells treated with AKBA, CDDP alone, or AKBA plus CDDP, the distributions of cell cycle were determined by flow cytometry (Fig. 9a). We found that AKBA increased visibly the percentages of G_0_/G_1_ phase in NC siRNA or p21 siRNA groups, and CDDP arrested cell cycle at G_2_/M phase. After the cotreatment of AKBA and CDDP, the percentages of G_0_/G_1_ phase were elevated and the proportions of G_2_/M phase were declined, compared with CDDP alone. Furthermore, there were lower percentages of G_0_/G_1_ phase of A549 cells treated with AKBA in p21 siRNA group than in NC siRNA group. After treatment of AKBA, the frequencies of S phase of A549 in NC siRNA group were markedly decreased, compared with control group; however, after treated with AKBA and transfected p21 siRNA, the proportions of S phase were significantly increased in A549 cells (Fig. 9b–d). In this study, we also investigated the effects of knockdown of p21 on cell regulators in A549 cells treated with two drugs alone or in combination. Next, we examined the effects of combination treatment on the expressions of G_0_/G_1_ phase associated proteins including cyclin A2, cyclin E1, cyclin D2, CDK2, and CDK4 by western blotting analysis. As shown in Fig. 9e, the expression of p21 was increased by treatment of CDDP alone, or in combination, and then was decreased after transfection of p21 siRNA, suggesting that we could continue to investigate the function effects of knockdown of p21 on other cell cycle proteins. Our results showed that AKBA inhibited the expressions of cyclin A2, cyclin D2, CDK2, and CDK4, CDDP increased the expressions of CDK2 and CDK4 proteins, and the cotreatment with AKBA and CDDP suppressed the expressions of G_0_/G_1_ phase associated protein cyclin A2, CDK2, and CDK4, compared with CDDP alone in NC siRNA group. When transfected by p21 siRNA, we found that compared with NC siRNA group, AKBA in combination with CDDP upregulated the expressions of cyclin A2, CDK2, and CDK4 proteins (Fig. 9f–j), and downregulated the expression of p21 protein (Fig. 9k), which explained that AKBA enhanced the sensitivity of CDDP to A549 through arresting cell cycle at G_0_/G_1_ phase via p21-dependent signaling pathway.

### AKBA enhanced the sensitivity of CDDP through apoptosis induction via p21-dependent signaling pathway in A549 cells

**Fig. 10:**
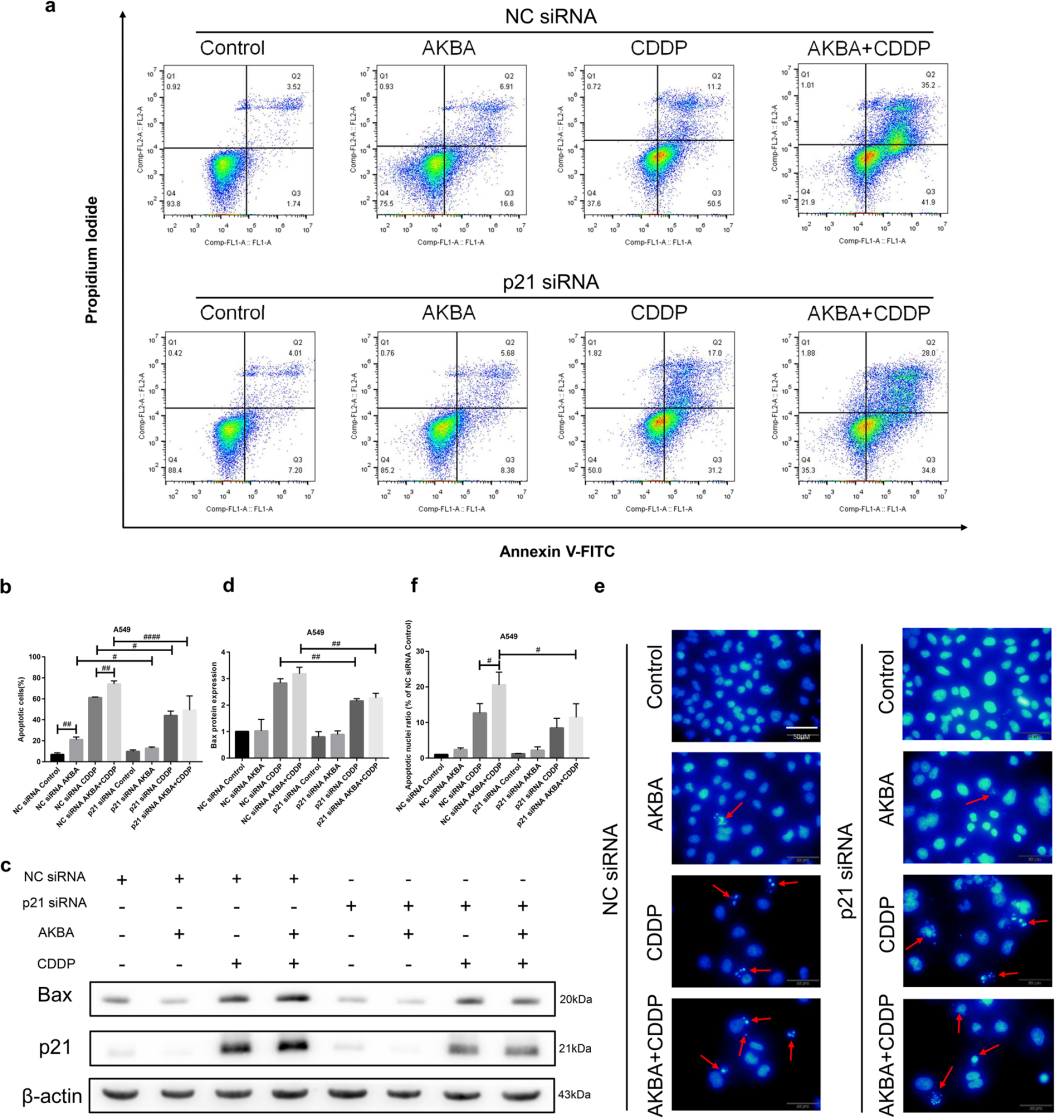
AKBA enhanced the sensitivity of CDDP (AKBA 10 μg/ml, CDDP 2 μg/ml) through apoptosis induction via p21-dependent signaling pathway in A549 cells. **a** Flow cytometry showed the apoptotic cell percentages after treatment of AKBA, CDDP alone, or in combination, in NC siRNA and p21 siRNA groups. **b** Histogram showing the apoptotic cell percentages of A549 cells and relative statistical analysis. **c**, **d** Bax and p21 protein expressions were determined by western blotting assay after knockdown of p21 in A549 cells treated with AKBA, CDDP alone, or in combination and relative statistical analysis. **e**, **f** Morphological alterations of the apoptotic nuclei of A549 and relative statistical analysis in each group. Scale bar = 50 μM. Data are represented as the mean ± SD of 3 independent experiments. ^#^*P* < 0.05, ^##^*P* < 0.01, ^####^*P* < 0.0001

To further investigate the molecular mechanism of apoptosis induction in A549 cells treated with AKBA, CDDP alone, or in combination, we used the A549 cells transfected by p21 siRNA to explore the effects of knockdown of p21 on cellular apoptosis. The percentages of apoptotic cells were determined by flow cytometry in NC siRNA and p21 siRNA groups (Fig. 10a). In this study, AKBA and CDDP both increased the proportions of apoptotic cells of A549 in NC siRNA group, and the combination treatment of AKBA and CDDP further elevated the frequencies of apoptotic cells of A549 in NC siRNA group. Interestingly, there were less apoptotic percentages of A549 cells treated with AKBA, CDDP alone, or AKBA plus CDDP in p21 siRNA group than in NC siRNA group, which demonstrated that AKBA strengthened the sensitivity of CDDP to A549 cells through apoptosis induction via p21-dependent signaling pathway (Fig. 10b). In addition, effects of cotreatment with AKBA and CDDP on apoptosis in A549 cells transfected by p21 siRNA were further shown by using western blotting analysis. As shown in Fig. 10c and d, the expression of Bax of A549 cells transfected by p21 siRNA was decreased after treatment of CDDP alone or AKBA plus CDDP compared with A549 cells transfected by NC siRNA, suggesting that knockdown of p21 decreased the percentages of apoptotic cells and these two drugs exerted anti-cancer effects via p21-depdent signaling pathway. Our results of immunofluorescence experiments also showed that after cotreatment with AKBA and CDDP, the ratio of apoptotic nuclei was decreased in A549 cells transfected by p21 siRNA, compared with NC siRNA group (Fig. 10e, f).

### AKBA enhanced the sensitivity of CDDP through autophagy suppression via p21-dependent signaling pathway in A549 cells

**Fig. 11:**
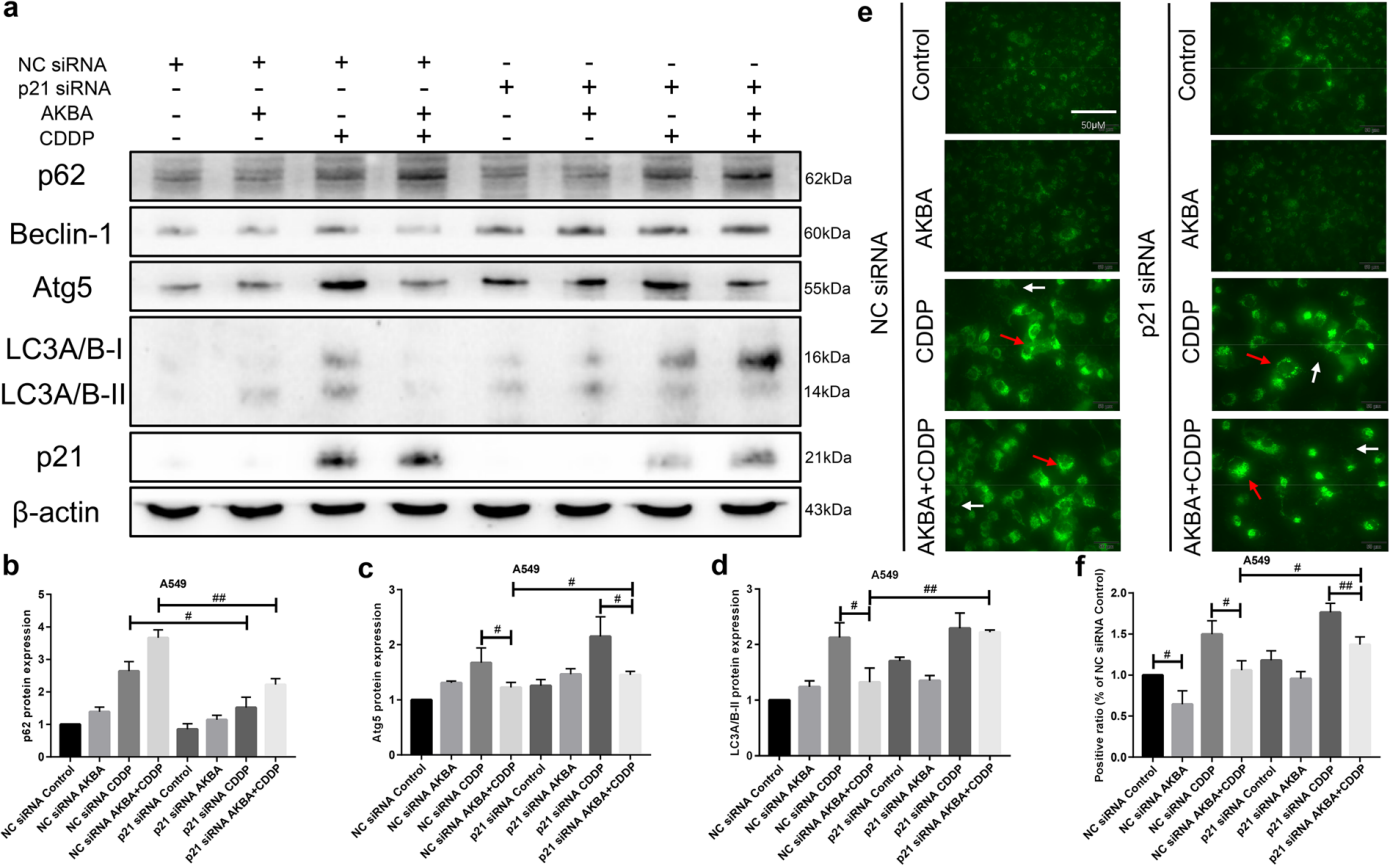
AKBA enhanced the sensitivity of CDDP (AKBA 10 μg/ml, CDDP 2 μg/ml) through autophagy suppression via p21-dependent signaling pathway in A549 cells. **a** p62, Atg5, Beclin-1, LC3A/B, and p21 protein expressions were measured by western blotting assay with or without knockdown of p21 in A549 cells treated with AKBA, CDDP alone, or in combination. **b**, **c**, **d** Histogram showing the level of p62, Atg5, and LC3A/B-II proteins and relative statistical analysis. **e** Representative images of the formation of autolysosome measured by immunofluorescence in A549. White arrow indicates negative autophagosome cell and the red indicates positive autophagsome cell. Scale bar = 50 μM. **f** Quantification of the positive phagosomes in A549. Data were represented as the mean ± SD of 3 independent experiments

According to the previous results, we knew that AKBA could strengthen the sensitivity of CDDP through suppressing autophagy. Hence, to study deeply the mechanism of chemo-sensitization, we selected A549 cells with knockdown of p21 to detect the expressions of autophagy associated proteins by using western blotting assay. As shown in Fig. 11a, we used western blotting assay to examine the expressions of autophagy proteins, finding that the expressions of Atg5, Beclin-1, and LC3A/B proteins in A549 cells transfected by p21 siRNA were elevated significantly after combination treatment of AKBA and CDDP, compared with NC siRNA group. Furthermore, the protein expression of p62 was upregulated after cotreatment with AKBA and CDDP in NC siRNA group, but it was downregulated after transfection of p21 siRNA (Fig. 11b, c, d). In addition, our results of immunofluorescence (DALGreen staining) showed that AKBA combined with CDDP reduced the ratio of positive autolysosome compared with CDDP alone. Furthermore, we found that compared with NC siRNA group, the ratio of the positive autolysosome formation of A549 cells treated with AKBA in combination with CDDP was increased in p21 siRNA groups (Fig. 11e, f). These evidences predicted that AKBA enhanced the sensitivity of CDDP to A549 by autophagy suppression via p21-dependent signaling pathway.

## Discussion

Cisplatin (CDDP) is the basic drug for chemotherapy of advanced NSCLC patients. However, the drug resistance and toxicity of CDDP limit its therapeutic effects and clinical practice in NSCLC patients (Siddik 2003). Therefore, the novel synergistic therapy needs to reduce the toxicity of cisplatin and increase its sensitivity. Recently, the active ingredients of natural plants, such as camptothecin and paclitaxel, have received extensive attention for their anti-tumor effects, and their combination with cisplatin has become the standard treatment for lung cancer, indicating that traditional Chinese herbal plants have a broad application prospect. AKBA, a pentacyclic triterpenoid from frankincense, has been shown to have anti-tumor effects in numerous studies, including glioblastoma (Li et al., 2018) and prostate cancer (Lu et al. 2008). In our previous study, we found that AKBA could exert anti-tumor effects in human NSCLC cell lines (Lv et al. 2020). However, whether or not AKBA enhanced the sensitivity of CDDP to NSCLC cell lines is still clearly unknown. In this study, we attempted to expound the synergistic effects of AKBA and CDDP on NSCLC cell lines and explore the possible mechanism of action in detail.

In cancer chemotherapy, it is of utmost importance to prevent the side effects of anti-cancer agents (Oun et al. 2018). Recent studies showed that AKBA attenuated oxidative glutamate toxicity in neuron-like cell lines (Rajabian et al. 2020) and can effectively protect kidney against interstitial fibrosis (Liu et al. 2018). The results of CCK8 assay, colony formation, and western blotting of BEAS-2B cells in our study also showed that AKBA could exert protection effects for normal cells. Our results of CCK8 assay in present study showed that AKBA enhanced the suppression effects of CDDP on cell viability in human NSCLC cell lines, but could weaken the cytotoxic effects of CDDP on cell viability in human normal lung epithelial (BEAS-2B) cells. Additionally, AKBA in combination with CDDP inhibited significantly clone formation of A549 and H1299 cells compared with either treatment with one alone. These evidences indicated that AKBA might be an effective chemotherapeutic sensitizer to human NSCLC cell lines.

On the basis of synergistic effects of two drugs on cell viability and clone formation of human NCSLC cell lines, we attempted to investigate cotreatment effects of AKBA and CDDP on cell cycle. Previous studies have shown that AKBA inhibited cell proliferation and arrested cell cycle in numerous cancers, such as leukemia (Hoernlein et al. 1999; Huang et al. 2000) and colon cancer (Yadav et al. 2012). In our study, AKBA arrested cell cycle at G_0_/G_1_ phase and decreased the percentages of S phase in A549. CDDP leaded to cell cycle arrest at G_2_/M phase. With the combination of AKBA and CDDP, the percentages of G_0_/G_1_ phase were elevated but the percentages of G_2_/M phase were decreased, compared with CDDP alone. Excessive proliferation is a common feature of most malignant tumors, in which cell cycle disorder is the important cause of tumorigenesis (Hanahan and Weinberg 2011). The center of cell cycle drive is the cyclin-dependent kinases (CDKs), combined with the cyclin proteins. In this study, we used western blotting assay to test the expression of CDK4 protein that is coupled with cyclin D, and examine the expression levels of cyclin A and cyclin E that bind to CDK2 protein. Then, the results showed that AKBA in combination with CDDP inhibited the expression of G_0_/G_1_ phase associated proteins, including CDK4, cyclin A, and cyclin E, compared with CDDP alone. These data were consistent with the previous flow cytometry, indicating that AKBA sensitized CDDP to NCSLC cells via inducing G_0_/G_1_ phase arrest through downregulating the relevant CDKs and cyclins. Cycle-dependent kinase inhibitors (CKI) that negatively regulate CDKs and cyclins play a significant role in cell cycle progression (Schafer 1998). P21 and p27 are currently accepted as potent universal CKI (Xiong et al. 1993). They physically interact with, and inhibit, the activity of cyclin-CDK1, -CDK2, and -CDK4/6 complexes, thus functioning as a regulator of cell cycle progression during the G_0_/G_1_ phases. In present study, we found that the mRNA expression levels of p21 and p27 were both increased after combination therapy of AKBA and CDDP, compared with CDDP alone. These results further illustrated that AKBA enhanced the sensitivity of CDDP to NSCLC cells though arresting cell cycle at G_0_/G_1_ phase.

Apoptosis is a form of programmed cell death that leads to the orderly and efficient removal of damaged cells, such as those resulting from DNA damage or during development (Pistritto et al. 2016). CDDP, as an apoptotic inducer that causes DNA-damaging (Rebillard et al. 2008), is widely used in treatment of various cancers, especially lung cancer. In our study, CDDP significantly increased the number of apoptotic cells in NSCLC cells. With the combination of CDDP and AKBA, the percentages of apoptosis were further elevated, which indicated that AKBA could enhance the sensitivity of CDDP on apoptosis induction in NSCLC cells. Studies showed that the Bcl-2 family of proteins control and regulate apoptotic mitochondrial events (Cory and Adams 2002). Targeting Bcl-2 family proteins is an effective approach to improving tumor sensitivity to CDDP on apoptosis induction (El-Daly et al. 2019; Li et al. 2015). Here, Bax as a pro-apoptotic protein of Bcl-2 family was determined by western blotting in this study, showing that AKBA in combination with CDDP increased the expression level of Bax protein compared with control group. We also found that after cotreatment of AKBA and CDDP, the expression of anti-apoptotic protein Bcl-xl was decreased in A549 cells, compared with CDDP alone. Additionally, DAPI staining assay showed that there were higher apoptotic nuclei ratio in combination group than in single treatment group. These results were consistent with the previous flow cytometry results, and all these data indicated that AKBA sensitized CDDP through increasing apoptosis in A549 cells, showing that the combination of AKBA and CDDP is a promising strategy for chemotherapy of NCSCL patients that are resistant to CDDP.

Autophagy allows energy supply during starvation, thus has been defined as a protective mechanism (Lum et al. 2005; White 2015). In the treatment of CDDP-based chemotherapy of NSCLC patients, the occurrence of autophagy is the significant reason of treatment failure or chemotherapy resistance (Levine and Kroemer 2008; Wu et al. 2015). A previous study demonstrated that upregulation of autophagy leads to CDDP resistance in NSCLC cells (Ren et al. 2010). Furthermore, recent studies showed that inhibition of autophagy enhanced the sensitivity of CDDP in numerous cancers, including ovarian cancer (Wan et al. 2018) and lung cancer(Chen et al. 2018), which indicated that debilitation of autophagy may be an effective approach to enhancing chemo-sensitivity in NSCLC (Sui et al. 2013). Our results in this study showed that CDDP enhanced autophagy because of the upregulation of LC3A/B protein and the increasing ratio of positive autolysosome in CDDP group, compared with control group. In addition, after cotreatment of CDDP and AKBA, the expression level of LC3A/B protein and the ratio of positive autolysosome were decreased in A549 cells, compared with CDDP alone. These evidences demonstrated that AKBA enhanced the sensitivity of CDDP to NSCLC cells via inhibiting autophagy.

P21, a cycle-dependent kinase inhibitor, has been reported to participate in the regulation of cell cycle (Dutto et al. 2015), apoptosis (Deng et al. 2016; Gartel and Tyner 2002), and autophagy (Capparelli et al. 2012; Fujiwara et al. 2008) in cancer. Recently, targeting therapy of p21 has been a novel approach to treating tumors (Moussa et al. 2019), and functional loss or downregulation of p21 has been suggested to mediate a drug-resistant phenotype following cancer therapy (Wei et al. 2010). Studies showed that targeting p21 can enhance the chemo-sensitivity of CDDP (Xu et al. 2016), and the upregulation of p21 increased the cisplatin cytotoxicity in NSCLC cells (Wei et al. 2010). In this study, we found that the mRNA expression level of p21 were increased after combination therapy of AKBA and CDDP, compared with CDDP alone, which possibly suggested that AKBA enhanced the sensitivity of CDDP via the upregulation of p21. In addition, our results of western blotting analysis found that AKBA in combination with CDDP suppressed the expressions of cyclin A2, CDK2, and CDK4, compared with CDDP alone, thereby arresting cell cycle at G_0_/G_1_ phase; however, the expression levels of these proteins were increased after downregulation of p21 in A549 cells treated with two drugs. Cell cycle distribution determined by flow cytometry showed that after treatment of AKBA, knockdown of p21 decreased the percentages of G_0_/G_1_ phase, negatively controlled the cyclins and proteins, thus to drive cell cycle from G_0_/G_1_ to S phase, and increased the proportions of S phase, suggesting that p21 is a promising therapeutic molecular target for cycle arrest treatment with AKBA, and treatment of AKBA further enhanced the chemo-sensitivity of CDDP to NSCLC A549 cells. We also found that AKBA, CDDP alone, or in combination all increased the apoptotic cells by flow cytometry, but after knockdown of p21, the percentages of apoptotic cells were obviously decreased in A549 cells, which indicated that p21 promoted apoptosis in NSCLC cells. Although how p21 promotes apoptosis exactly is not clear, studies showed that it might depend on both p53-dependent and p53-independent upregulation of the pro-apoptotic protein Bax (Gartel 2005; Kang et al. 1999). In present study, when A549 cells treated with CDDP or CDDP plus AKBA, the expression level of Bax protein of A549 was downregulated after knockdown of p21, and it was consistent with previous results, indicated that AKBA enhanced the sensitivity of CDDP to induce apoptosis through upregulation of Bax protein via p21-dependent pathway. Recent studies showed that upregulation of p21 inhibits autophagy in cisplatin-resistant cancer cells (Wei et al. 2017), which may make p21 become a promising therapeutic molecular target for reducing chemotherapeutic resistance caused by autophagy. Hence, we downregulated the expression level of p21 in A549 cells, and then investigated whether or not knockdown of p21 exerted the acceleration effects on autophagy. Results of western blotting analysis showed that compared with single treatment of CDDP, AKBA in combination with CDDP inhibited the expressions of Atg5 and LC3A/B-II proteins, and upregulated the expression of p62 protein; however, after downregulation of p21, the expressions of downregulated proteins Atg5 and LC3A/B-II were increased and the expression of upregulated protein p62 was decreased in A549 cells. These data indicated AKBA sensitized the effects of CDDP on autophagy via targeting p21. Although we expound minutely mechanism of action that AKBA enhanced the sensitivity of CDDP to NSCLC cells via targeting p21 pathway, this still needs an animal model and cisplatin-resistant cell lines to further identify our conclusion in vivo and in vitro.

## Conclusion

In summary, AKBA in combination with CDDP strengthened the anti-tumor effects of CDDP on cell viability in human NSCLC cell lines but decreased the cytotoxic effects of CDDP on cell viability in human normal lung epithelial cell line BEAS-2B, compared with single treatment of CDDP. Furthermore, AKBA enhanced the sensitivity of CDDP on cell cycle arrest, apoptosis induction, and autophagy inhibition via targeting p21-dependent signaling pathway. Our data indicated that AKBA, a bioactive compound from frankincense, in combination with CDDP, might serve as a new therapeutic regimen for NSCLC.

## Electronic supplementary material

ESM 1 (DOCX 3220 kb)

## Abbreviations

NSCLC: Non-small cell lung cancer
AKBA: Acetyl-11-keto-β-boswellic acid
Cisplatin: CDDP
CKI: Cyclin-dependent kinase inhibitor
CDK: Cyclin-dependent kinases

## References

[CR1] Ammon HP (2016). Boswellic acids and their role in chronic inflammatory diseases. Adv Exp Med Biol.

[CR2] Belani CP (2002). Chemotherapy regimens in advanced non-small-cell lung cancer: recent randomized trials. Clin Lung Cancer.

[CR3] Bray F, Ferlay J, Soerjomataram I, Siegel RL, Torre LA, Jemal A (2018). Global cancer statistics 2018: GLOBOCAN estimates of incidence and mortality worldwide for 36 cancers in 185 countries. CA Cancer J Clin.

[CR4] Capparelli C, Chiavarina B, Whitaker-Menezes D, Pestell TG, Pestell RG, Hulit J, Ando S, Howell A, Martinez-Outschoorn UE, Sotgia F, Lisanti MP (2012). CDK inhibitors (p16/p19/p21) induce senescence and autophagy in cancer-associated fibroblasts, “fueling” tumor growth via paracrine interactions, without an increase in neo-angiogenesis. Cell Cycle.

[CR5] Chen Y, Li J, Chen S, Zhang Y, Hu Y, Zhang G, Yan X, Jiao S (2017). Nab-paclitaxel in combination with cisplatin versus docetaxel plus cisplatin as first-line therapy in non-small cell lung cancer. Sci Rep.

[CR6] Chen J, Zhang L, Zhou H, Wang W, Luo Y, Yang H, Yi H (2018). Inhibition of autophagy promotes cisplatin-induced apoptotic cell death through Atg5 and Beclin 1 in A549 human lung cancer cells. Mol Med Rep.

[CR7] Cory S, Adams JM (2002). The Bcl2 family: regulators of the cellular life-or-death switch. Nat Rev Cancer.

[CR8] Deng S, Tang S, Dai C, Zhou Y, Yang X, Li D, Xiao X (2016). P21(Waf1/Cip1) plays a critical role in furazolidone-induced apoptosis in HepG2 cells through influencing the caspase-3 activation and ROS generation. Food Chem Toxicol.

[CR9] Dutto I, Tillhon M, Cazzalini O, Stivala LA, Prosperi E (2015). Biology of the cell cycle inhibitor p21(CDKN1A): molecular mechanisms and relevance in chemical toxicology. Arch Toxicol.

[CR10] Eastham JA, Hall SJ, Sehgal I, Wang J, Timme TL, Yang G, Connell-Crowley L, Elledge SJ, Zhang WW, Harper JW, Et A (1995). In vivo gene therapy with p53 or p21 adenovirus for prostate cancer. Cancer Res.

[CR11] El-Daly SM, Gouhar SA, Gamal-Eldeen AM, Abdel HF, Ashour MN, Hassan NS. Synergistic effect of alpha-solanine and cisplatin induces apoptosis and enhances cell cycle arrest in human hepatocellular carcinoma cells. Anti Cancer Agents Med Chem. 2019. 10.2174/1871520619666190930123520 31566136

[CR12] Fujiwara K, Daido S, Yamamoto A, Kobayashi R, Yokoyama T, Aoki H, Iwado E, Shinojima N, Kondo Y, Kondo S (2008). Pivotal role of the cyclin-dependent kinase inhibitor p21WAF1/CIP1 in apoptosis and autophagy. J Biol Chem.

[CR13] Gartel AL (2005). The conflicting roles of the cdk inhibitor p21(CIP1/WAF1) in apoptosis. Leuk Res.

[CR14] Gartel AL, Tyner AL (2002). The role of the cyclin-dependent kinase inhibitor p21 in apoptosis. Mol Cancer Ther.

[CR15] Gridelli C, Rossi A, Carbone DP, Guarize J, Karachaliou N, Mok T, Petrella F, Spaggiari L, Rosell R (2015). Non-small-cell lung cancer. Nat Rev Dis Primers.

[CR16] Hanahan D, Weinberg RA (2011). Hallmarks of cancer: the next generation. Cell..

[CR17] Harper JW, Adami GR, Wei N, Keyomarsi K, Elledge SJ (1993). The p21 Cdk-interacting protein Cip1 is a potent inhibitor of G1 cyclin-dependent kinases. Cell..

[CR18] Hoernlein RF, Orlikowsky T, Zehrer C, Niethammer D, Sailer ER, Simmet T, Dannecker GE, Ammon HP (1999). Acetyl-11-keto-beta-boswellic acid induces apoptosis in HL-60 and CCRF-CEM cells and inhibits topoisomerase I. J Pharmacol Exp Ther.

[CR19] Huang MT, Badmaev V, Ding Y, Liu Y, Xie JG, Ho CT (2000). Anti-tumor and anti-carcinogenic activities of triterpenoid, beta-boswellic acid. Biofactors..

[CR20] Jekunen AP, Christen RD, Shalinsky DR, Howell SB (1994). Synergistic interaction between cisplatin and taxol in human ovarian carcinoma cells in vitro. Br J Cancer.

[CR21] Ji SQ, Zhang YX, Yang BH (2017). UBR5 promotes cell proliferation and inhibits apoptosis in colon cancer by destablizing P21. Pharmazie..

[CR22] Kang KH, Kim WH, Choi KH (1999). p21 promotes ceramide-induced apoptosis and antagonizes the antideath effect of Bcl-2 in human hepatocarcinoma cells. Exp Cell Res.

[CR23] Khan MA, Ali R, Parveen R, Najmi AK, Ahmad S (2016). Pharmacological evidences for cytotoxic and antitumor properties of Boswellic acids from Boswellia serrata. J Ethnopharmacol.

[CR24] Lazareva NF, Baryshok VP, Lazarev IM. Silicon-containing analogs of camptothecin as anticancer agents. Arch Pharm (Weinheim). 2018;351. 10.1002/ardp.201700297 29239010

[CR25] Levine B, Kroemer G (2008). Autophagy in the pathogenesis of disease. Cell..

[CR26] Li X, Huang JM, Wang JN, Xiong XK, Yang XF, Zou F (2015). Combination of chrysin and cisplatin promotes the apoptosis of Hep G2 cells by up-regulating p53. Chem Biol Interact.

[CR27] Li W, Liu J, Fu W, Zheng X, Ren L, Liu S, Wang J, Ji T, Du G (2018). 3-O-acetyl-11-keto-beta-boswellic acid exerts anti-tumor effects in glioblastoma by arresting cell cycle at G2/M phase. J Exp Clin Cancer Res.

[CR28] Li Y, Huang J, Zeng B, Yang D, Sun J, Yin X, Lu M, Qiu Z, Peng W, Xiang T, Li H, Ren G (2018). PSMD2 regulates breast cancer cell proliferation and cell cycle progression by modulating p21 and p27 proteasomal degradation. Cancer Lett.

[CR29] Liu M, Liu T, Shang P, Zhang Y, Liu L, Liu T, Sun S (2018). Acetyl-11-keto-beta-boswellic acid ameliorates renal interstitial fibrosis via Klotho/TGF-beta/Smad signalling pathway. J Cell Mol Med.

[CR30] Lu M, Xia L, Hua H, Jing Y (2008). Acetyl-keto-beta-boswellic acid induces apoptosis through a death receptor 5-mediated pathway in prostate cancer cells. Cancer Res.

[CR31] Lum JJ, Bauer DE, Kong M, Harris MH, Li C, Lindsten T, et al. Growth factor regulation of autophagy and cell survival in the absence of apoptosis. Cell. 2005;120. 10.1016/j.cell.2004.11.046 15680329

[CR32] Lv M, Shao S, Zhang Q, Zhuang X, Qiao T (2020). Acetyl-11-keto-beta-boswellic acid exerts the anti-cancer effects via cell cycle arrest, apoptosis induction and autophagy suppression in non-small cell lung cancer cells. Onco Targets Ther.

[CR33] Matsumoto M, Nakajima W, Seike M, Gemma A, Tanaka N (2016). Cisplatin-induced apoptosis in non-small-cell lung cancer cells is dependent on Bax- and Bak-induction pathway and synergistically activated by BH3-mimetic ABT-263 in p53 wild-type and mutant cells. Biochem Biophys Res Commun.

[CR34] Meng Z, Lv Q, Lu J, Yao H, Lv X, Jiang F, et al. Prodrug strategies for paclitaxel. Int J Mol Sci. 2016;17. 10.3390/ijms17050796 PMC4881612 27223283

[CR35] Mitsudomi T, Morita S, Yatabe Y, Negoro S, Okamoto I, Tsurutani J, Seto T, Satouchi M, Tada H, Hirashima T, Asami K, Katakami N, Takada M, Yoshioka H, Shibata K, Kudoh S, Shimizu E, Saito H, Toyooka S, Nakagawa K, Fukuoka M (2010). Gefitinib versus cisplatin plus docetaxel in patients with non-small-cell lung cancer harbouring mutations of the epidermal growth factor receptor (WJTOG3405): an open label, randomised phase 3 trial. Lancet Oncol.

[CR36] Moussa RS, Park KC, Kovacevic Z, Richardson DR (2019). Ironing out the role of the cyclin-dependent kinase inhibitor, p21 in cancer: novel iron chelating agents to target p21 expression and activity. Free Radic Biol Med.

[CR37] Olaussen KA, Dunant A, Fouret P, Brambilla E, Andre F, Haddad V, Taranchon E, Filipits M, Pirker R, Popper HH, Stahel R, Sabatier L, Pignon JP, Tursz T, Le Chevalier T, Soria JC (2006). DNA repair by ERCC1 in non-small-cell lung cancer and cisplatin-based adjuvant chemotherapy. N Engl J Med.

[CR38] Oun R, Moussa YE, Wheate NJ (2018). The side effects of platinum-based chemotherapy drugs: a review for chemists. Dalton Trans.

[CR39] Park B, Sung B, Yadav VR, Cho SG, Liu M, Aggarwal BB (2011). Acetyl-11-keto-beta-boswellic acid suppresses invasion of pancreatic cancer cells through the downregulation of CXCR4 chemokine receptor expression. Int J Cancer.

[CR40] Pistritto G, Trisciuoglio D, Ceci C, Garufi A, D'Orazi G (2016). Apoptosis as anticancer mechanism: function and dysfunction of its modulators and targeted therapeutic strategies. Aging (Albany NY).

[CR41] Qin LF, Ng IO (2001). Exogenous expression of p21(WAF1/CIP1) exerts cell growth inhibition and enhances sensitivity to cisplatin in hepatoma cells. Cancer Lett.

[CR42] Rajabian A, Sadeghnia HR, Hosseini A, Mousavi SH, Boroushaki MT (2020). 3-Acetyl-11-keto-beta-boswellic acid attenuated oxidative glutamate toxicity in neuron-like cell lines by apoptosis inhibition. J Cell Biochem.

[CR43] Rebillard A, Lagadic-Gossmann D, Dimanche-Boitrel MT (2008). Cisplatin cytotoxicity: DNA and plasma membrane targets. Curr Med Chem.

[CR44] Ren JH, He WS, Nong L, Zhu QY, Hu K, Zhang RG, Huang LL, Zhu F, Wu G (2010). Acquired cisplatin resistance in human lung adenocarcinoma cells is associated with enhanced autophagy. Cancer Biother Radiopharm.

[CR45] Rowinsky EK, Donehower RC (1995). Paclitaxel (taxol). N Engl J Med.

[CR46] Scagliotti GV, Parikh P, von Pawel J, Biesma B, Vansteenkiste J, Manegold C, Serwatowski P, Gatzemeier U, Digumarti R, Zukin M, Lee JS, Mellemgaard A, Park K, Patil S, Rolski J, Goksel T, de Marinis F, Simms L, Sugarman KP, Gandara D (2008). Phase III study comparing cisplatin plus gemcitabine with cisplatin plus pemetrexed in chemotherapy-naive patients with advanced-stage non-small-cell lung cancer. J Clin Oncol.

[CR47] Schafer KA (1998). The cell cycle: a review. Vet Pathol.

[CR48] Shamimi-Noori S, Yeow WS, Ziauddin MF, Xin H, Tran TL, Xie J, Loehfelm A, Patel P, Yang J, Schrump DS, Fang BL, Nguyen DM (2008). Cisplatin enhances the antitumor effect of tumor necrosis factor-related apoptosis-inducing ligand gene therapy via recruitment of the mitochondria-dependent death signaling pathway. Cancer Gene Ther.

[CR49] Shamma M, St GV (1974). Camptothecin. J Pharm Sci.

[CR50] Siddik ZH (2003). Cisplatin: mode of cytotoxic action and molecular basis of resistance. Oncogene..

[CR51] Silvestri R (2013). New prospects for vinblastine analogues as anticancer agents. J Med Chem.

[CR52] Sui X, Chen R, Wang Z, Huang Z, Kong N, Zhang M, Han W, Lou F, Yang J, Zhang Q, Wang X, He C, Pan H (2013). Autophagy and chemotherapy resistance: a promising therapeutic target for cancer treatment. Cell Death Dis.

[CR53] Sun CY, Zhu Y, Li XF, Wang XQ, Tang LP, Su ZQ, Li CY, Zheng GJ, Feng B (2018). Scutellarin increases cisplatin-induced apoptosis and autophagy to overcome cisplatin resistance in non-small cell lung cancer via ERK/p53 and c-met/AKT signaling pathways. Front Pharmacol.

[CR54] Syrovets T, Buchele B, Krauss C, Laumonnier Y, Simmet T (2005). Acetyl-boswellic acids inhibit lipopolysaccharide-mediated TNF-alpha induction in monocytes by direct interaction with IkappaB kinases. J Immunol.

[CR55] Takada Y, Ichikawa H, Badmaev V, Aggarwal BB (2006). Acetyl-11-keto-beta-boswellic acid potentiates apoptosis, inhibits invasion, and abolishes osteoclastogenesis by suppressing NF-kappa B and NF-kappa B-regulated gene expression. J Immunol.

[CR56] Ulukaya E, Ari F, Dimas K, Sarimahmut M, Guney E, Sakellaridis N, Yilmaz VT (2011). Cell death-inducing effect of novel palladium(II) and platinum(II) complexes on non-small cell lung cancer cells in vitro. J Cancer Res Clin Oncol.

[CR57] Wan B, Dai L, Wang L, Zhang Y, Huang H, Qian G, Yu T (2018). Knockdown of BRCA2 enhances cisplatin and cisplatin-induced autophagy in ovarian cancer cells. Endocr Relat Cancer.

[CR58] Wei J, Zhao J, Long M, Han Y, Wang X, Lin F, Ren J, He T, Zhang H (2010). p21WAF1/CIP1 gene transcriptional activation exerts cell growth inhibition and enhances chemosensitivity to cisplatin in lung carcinoma cell. BMC Cancer.

[CR59] Wei F, Jiang X, Gao HY, Gao SH (2017). Liquiritin induces apoptosis and autophagy in cisplatin (DDP)-resistant gastric cancer cells in vitro and xenograft nude mice in vivo. Int J Oncol.

[CR60] White E (2015). The role for autophagy in cancer. J Clin Invest.

[CR61] Wu HM, Jiang ZF, Ding PS, Shao LJ, Liu RY (2015). Hypoxia-induced autophagy mediates cisplatin resistance in lung cancer cells. Sci Rep.

[CR62] X P, Y Z, Z X (2009). Acetyl-11-keto-beta-boswellic acid inhibits prostate tumor growth by suppressing vascular endothelial growth factor receptor 2-mediated angiogenesis. Cancer Res.

[CR63] Xiong Y, Hannon GJ, Zhang H, Casso D, Kobayashi R, Beach D (1993). p21 is a universal inhibitor of cyclin kinases. Nature..

[CR64] Xu S, Huang H, Chen YN, Deng YT, Zhang B, Xiong XD, Yuan Y, Zhu Y, Huang H, Xie L, Liu X (2016). DNA damage responsive miR-33b-3p promoted lung cancer cells survival and cisplatin resistance by targeting p21(WAF1/CIP1). Cell Cycle.

[CR65] Yadav VR, Prasad S, Sung B, Gelovani JG, Guha S, Krishnan S, Aggarwal BB (2012). Boswellic acid inhibits growth and metastasis of human colorectal cancer in orthotopic mouse model by downregulating inflammatory, proliferative, invasive and angiogenic biomarkers. Int J Cancer.

[CR66] Zhang L, Chen J, Ning D, Liu Q, Wang C, Zhang Z, Chu L, Yu C, Liang HF, Zhang B, Chen X (2019). FBXO22 promotes the development of hepatocellular carcinoma by regulating the ubiquitination and degradation of p21. J Exp Clin Cancer Res.

