# Supplementary material for: Acetyl-11-keto-β-boswellic acid enhances the cisplatin sensitivity of non-small cell lung cancer cells through cell cycle arrest, apoptosis induction, and autophagy suppression via p21-dependent signaling pathway
Source: Cell Biol Toxicol. 2020 Jun 20;37(2):209–28. doi: 10.1007/s10565-020-09541-5 (PMC8012341; doi:10.1007/s10565-020-09541-5)
Supplement: Supplementary file 1 — (DOCX 3220 kb) [file 10565_2020_9541_MOESM1_ESM.docx]

**Verification of knockdown of p21 in mRNA and protein levels in A549 cells**

P21, a well-established cyclin-dependent kinase inhibitor (CKI), was elevated by treatment of AKBA or CDDP, and it was further increased in AKBA plus CDDP group. Therefore, to further explore the anti-tumor mechanism of the cotreatment of AKBA and CDDP, we transfected three different small interfering RNAs (siRNA) into A549 cells. Then, whether or not the mRNA expression level of p21 was reduced was verified by real-time fluorescence quantitative PCR and western blotting assay. We found that the mRNA and protein expression levels of p21 of A549 cells transfected by p21 376 siRNA were lowest among three different p21 siRNA transfection groups **(Fig. S1a, b, c)**. Hence, we selected the p21 siRNA with the best knockdown effect for subsequent experiments.

**
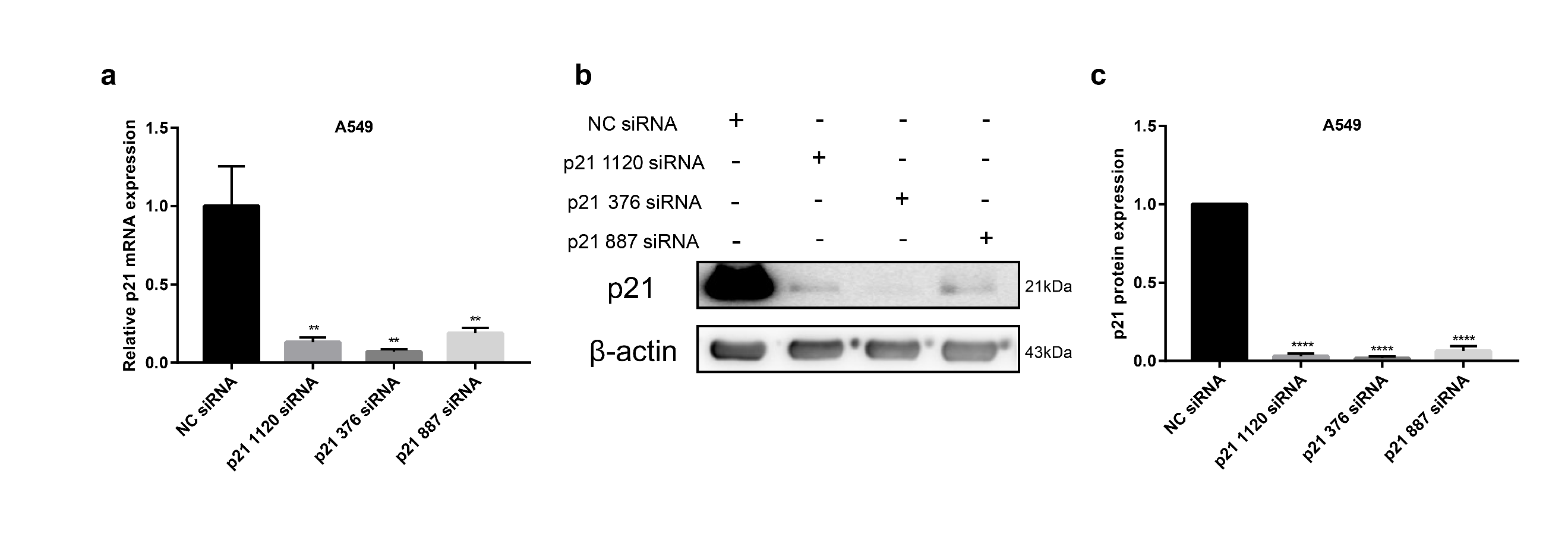
 Fig. S1** Verification of knockdown of p21 in mRNA and protein levels in A549 cells. **(a)** The mRNA expression level of p21 after transfection of three p21 siRNA. **(b)** The protein expression level of p21 after transfection of three p21 siRNA. **(c)** Histogram showing the level of p21 protein and relative statistical analysis after transfection of three p21 siRNA. Data were represented as the mean ± SD of 3 independent experiments, ** P <0.01, **** P < 0.0001, vs. NC siRNA group.

**The Effects of AKBA in combination with cisplatin on cell viability of NSCLC cell lines and BEAS-2B.**

To explore the appropriate concentration of AKBA in combination with CDDP, we used CCK8 assay to test the cell viability of NSCLC cell lines and BEAS-2B, finding that AKBA at 10 μg/ml reduced markedly the toxic effects of CDDP on BEAS-2B cells at 48 h, and 72 h, but enhanced the suppression effects of CDDP on the cell viability of A549 and H1299 cells **(Fig. S2a, b, c)**. Therefore, we choose AKBA at 10 μg/ml in combination with CDDP to treat the NSCLC cell lines and BEAS-2B cells of each group in subsequent experiment.


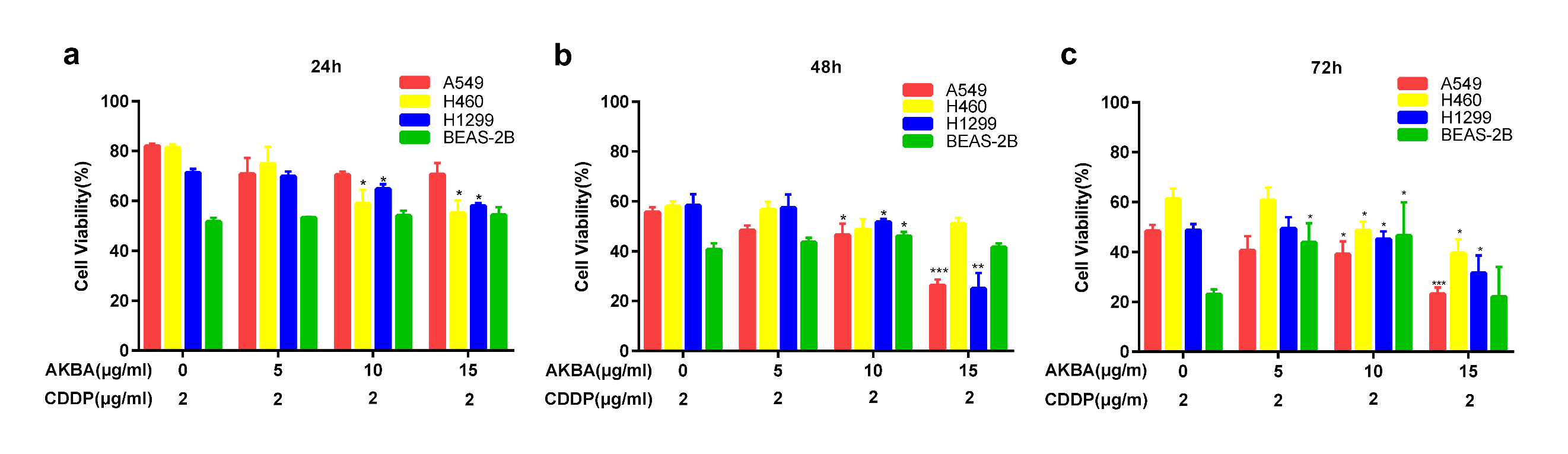


**Fig. S2** The Effects of AKBA in combination with cisplatin on cell viability of NSCLC cell lines and BEAS-2B. **(a, b, c)** AKBA reduced the toxic effect of cisplatin on BEAS-2B but enhanced the inhibition effects of cisplatin on NSCLC cell lines at 24 h, 48h, and 72h. Data were represented as the mean ± SD of 3 independent experiments, * P <0.05, ** P <0.01, *** P < 0.001, vs. 0 μg/ml AKBA + 2 μg/ml CDDP group.

**The effects of AKBA and CDDP on the colony formation and cellular morphology in BEAS-2B**

To further study the effects of AKBA combined with CDDP on cell proliferation, we employed colony formation assay to determine the ability of cell proliferation after AKBA, CDDP alone or both. The results showed that compared to control group, AKBA enhanced the colony number but CDDP alone or in combination with AKBA almost inhibited completely the colony formation in BEAS-2B cells. Specially, there were some adherent cells but no colonies in combined group **(Fig. S3a, b)**. As shown in **Fig. S3c**, we found that compared to control group, AKBA increased the cell number of BEAS-2B but CDDP decreased the cell number of BEAS-2B. Furthermore, AKBA in combination with CDDP increased the cell number of BEAS-2B compared to CDDP alone and two drugs hardly altered the cellular morphology in BEAS-2B cells.


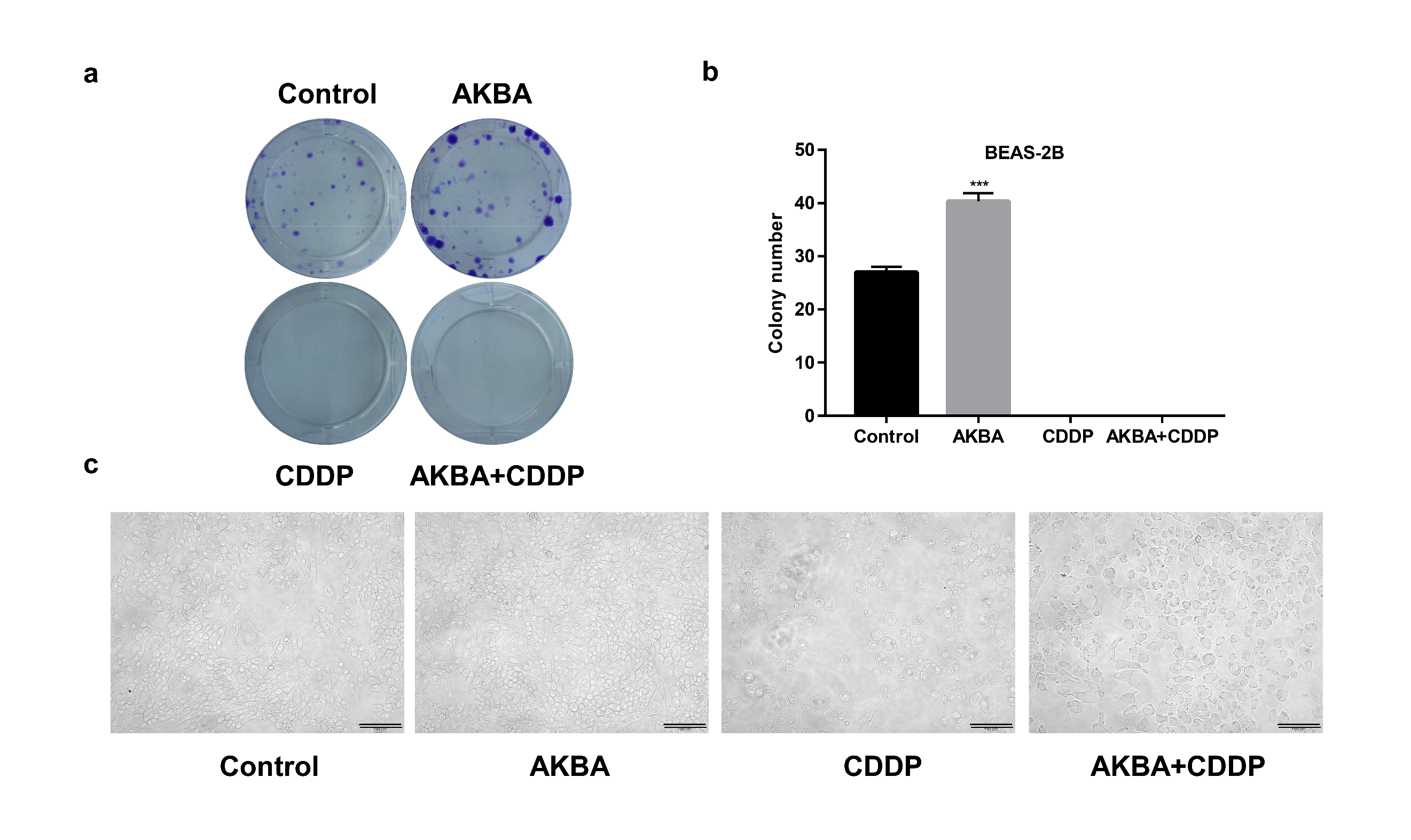


**Fig. S3** The effects of AKBA and CDDP on the colony formation and cellular morphology in BEAS-2B. **(a)** The effects of AKBA and CDDP on colony formation in BEAS-2B. **(b)** Histogram showing the colony number and relative statistical analysis. **(c)** The effects of AKBA and CDDP on cellular morphology in BEAS-2B. Scale bar=200 μM. Data were represented as the mean ± SD of 3 independent experiments, *** P < 0.001, vs. Control group.

**The effects of AKBA in combination with cisplatin on the regulators of cell cycle in BEAS-2B.**

To further investigate the effects of AKBA and CDDP on the regulators of cell cycle, we used western blotting assay to test the expression levels of the cell cycle associated proteins **(Fig. 4Sa).** The results of western blotting showed that AKBA in combination with CDDP increased the expressions of Cyclin A2 and p-cdc2 proteins compared to CDDP alone in BEAS-2B cells **(Fig. 4Sb, c)**. In addition, we found that the expressions of p27 and p21 proteins were upregulated by treatment of AKBA but were downregulated by treatment of CDDP compared to control group in BEAS-2B cells. However, AKBA in combination with CDDP elevated obviously the expressions of p27 and p21 proteins in BEAS-2B **(Fig. 4Sd, e, f)**.

**
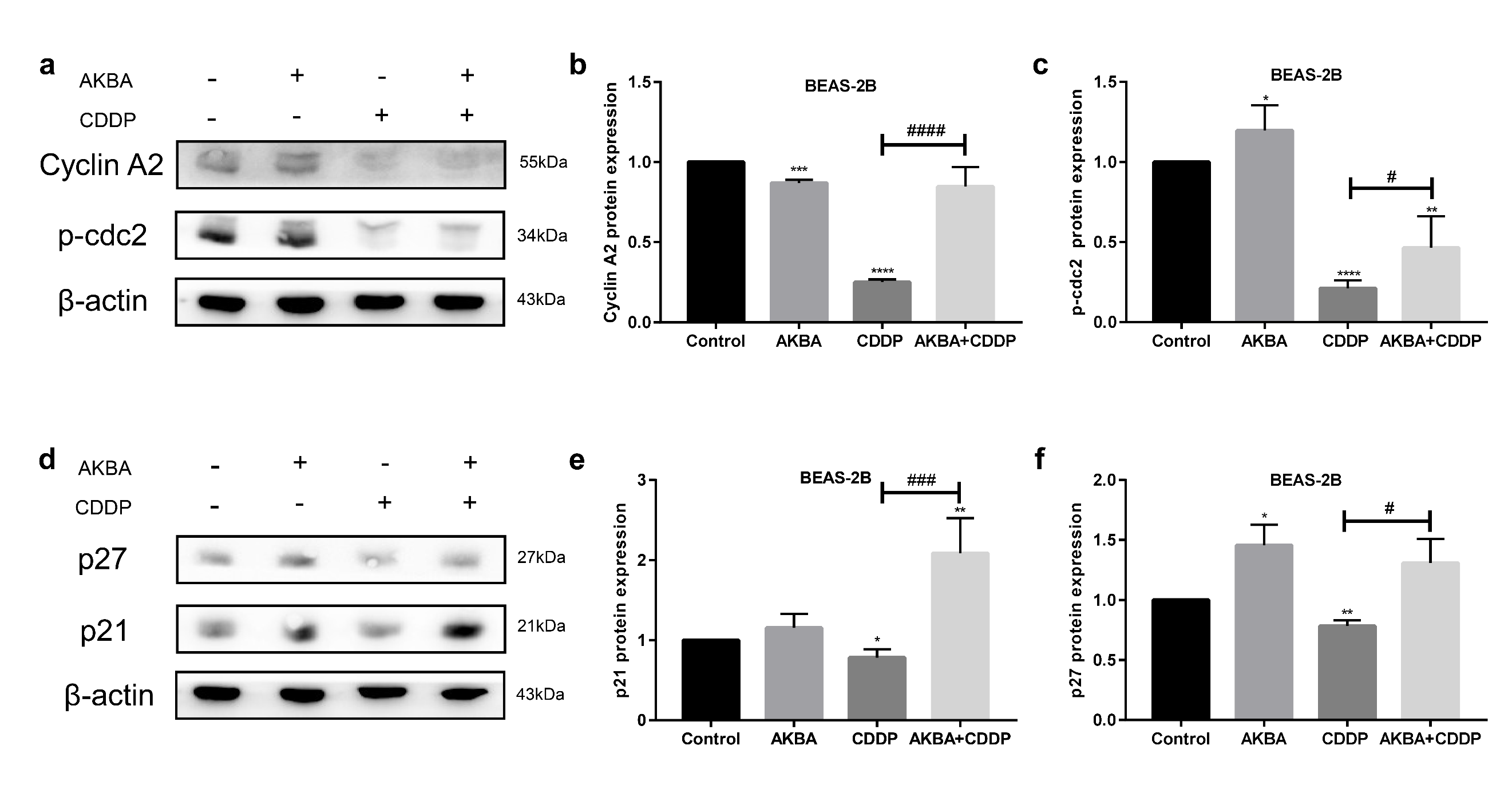
**

**Fig. S4** The effects of AKBA in combination with cisplatin on the regulator of cell cycle in BEAS-2B. **(a)** The expressions of cyclin A2 and p-cdc2 proteins were determined by western blotting assay in four groups. **(b)** Histogram showing the colony number and relative statistical analysis. **(c)** Histogram showing the level of cyclin A2 protein expression and relative statistical analysis. **(d)** Representative images of p27 and p21 proteins, using β-actin as internal control. **(e)** Histogram showing the level of p27 protein expression and relative statistical analysis. **(f)** Histogram showing the level of p21 protein expression and relative statistical analysis. Data were represented as the mean ± SD of 3 independent experiments, *P <0.05, ** P <0.01, *** P < 0.001, **** P < 0.0001, vs. Control group. # P < 0.05, ### P < 0.001, #### P < 0.0001.
